# Phase separation promotes a highly active oligomeric scaffold of the MLL1 core complex for regulation of histone H3K4 methylation

**DOI:** 10.1016/j.jbc.2023.105204

**Published:** 2023-09-01

**Authors:** Kevin E.W. Namitz, Scott A. Showalter, Michael S. Cosgrove

**Affiliations:** 1 Department of Biochemistry and Molecular Biology, State University of New York (SUNY) Upstate Medical University, Syracuse, New York, USA; 2 Department of Chemistry, Penn State University, University Park, Pennsylvania, USA

**Keywords:** histone methylation, phase separation, analytical ultracentrifugation, NMR spectroscopy, enzyme kinetics

## Abstract

Enzymes that regulate the degree of histone H3 lysine 4 (H3K4) methylation are crucial for proper cellular differentiation and are frequently mutated in cancer. The Mixed lineage leukemia (MLL) family of enzymes deposit H3K4 mono-, di-, or trimethylation at distinct genomic locations, requiring precise spatial and temporal control. Despite evidence that the degree of H3K4 methylation is controlled in part by a hierarchical assembly pathway with key subcomplex components, we previously found that the assembled state of the MLL1 core complex is not favored at physiological temperature. To better understand this paradox, we tested the hypothesis that increasing the concentration of subunits in a biomolecular condensate overcomes this thermodynamic barrier *via* mass action. Here, we demonstrate that MLL1 core complex phase separation stimulates enzymatic activity up to 60-fold but not primarily by concentrating subunits into droplets. Instead, we found that stimulated activity is largely due to the formation of an altered oligomeric scaffold that greatly reduces substrate *K*_*m*_. We posit that phase separation–induced scaffolding of the MLL1 core complex is a potential “switch-like” mechanism for spatiotemporal control of H3K4 methylation through the rapid formation or dissolution of biomolecular condensates within RNA Pol II transcription factories.

Patterns of histone H3 lysine 4 (H3K4) methylation contribute to cell identity and are frequently disrupted in disease. Several distinct but linked pathways involving histone lysine methyltransferases and demethylases coordinate to ensure the proper cell type–specific patterning of H3K4 methylation. In general, H3K4 di-methylation and trimethylation are enriched in the open reading frames (ORFs) and promoters of active genes (1, 2, 3), respectively, and are required to recruit nucleosome remodeling complexes that promote gene accessibility (4, 5, 6, 7, 8). H3K4 monomethylation is enriched at active gene enhancers and in the promoters of silenced genes (9, 10, 11, 12, 13). The molecular mechanisms that establish spatial and temporal control of the different degrees of H3K4 methylation are unknown.

In humans, the six members of the mixed lineage leukemia (MLL)/SET1 family (MLL1-4, SETd1a, b) are the major implementers of H3K4 methylation (14). Each possess a catalytic C-terminal Suppressor of Variegation, Enhancer of Zeste, Trithorax (SET) domain at the end of large (1000–2000aa) intrinsically disordered regions of unknown function (Figs. 1*A*, and S1; Table S6). The SET domain of MLL1 has been shown to catalyze H3K4 monomethylation (15), whereas multiple methylation requires interaction with a conserved subcomplex called WRAD_2_ (WDR5, RbBP5, Ash2L, DPY-30), forming the MLL1 core complex (MWRAD_2_) (15, 16) (Fig. 1*B*). In a previous investigation, we showed that the MLL1 core complex is hierarchically assembled from MW and RAD_2_ subcomplexes in an interaction that is highly concentration- and temperature-dependent (17). Intriguingly, we found that the disassembled state of the MLL1 core complex is favored at physiological temperature and that increased protein concentration partially overcomes this thermodynamic barrier for complex assembly. These results suggest a possible regulatory mechanism for spatial and temporal control of H3K4 methylation by concentration of subunits in biomolecular condensates, such as those found in transcription factories (18).

**Figure 1:**
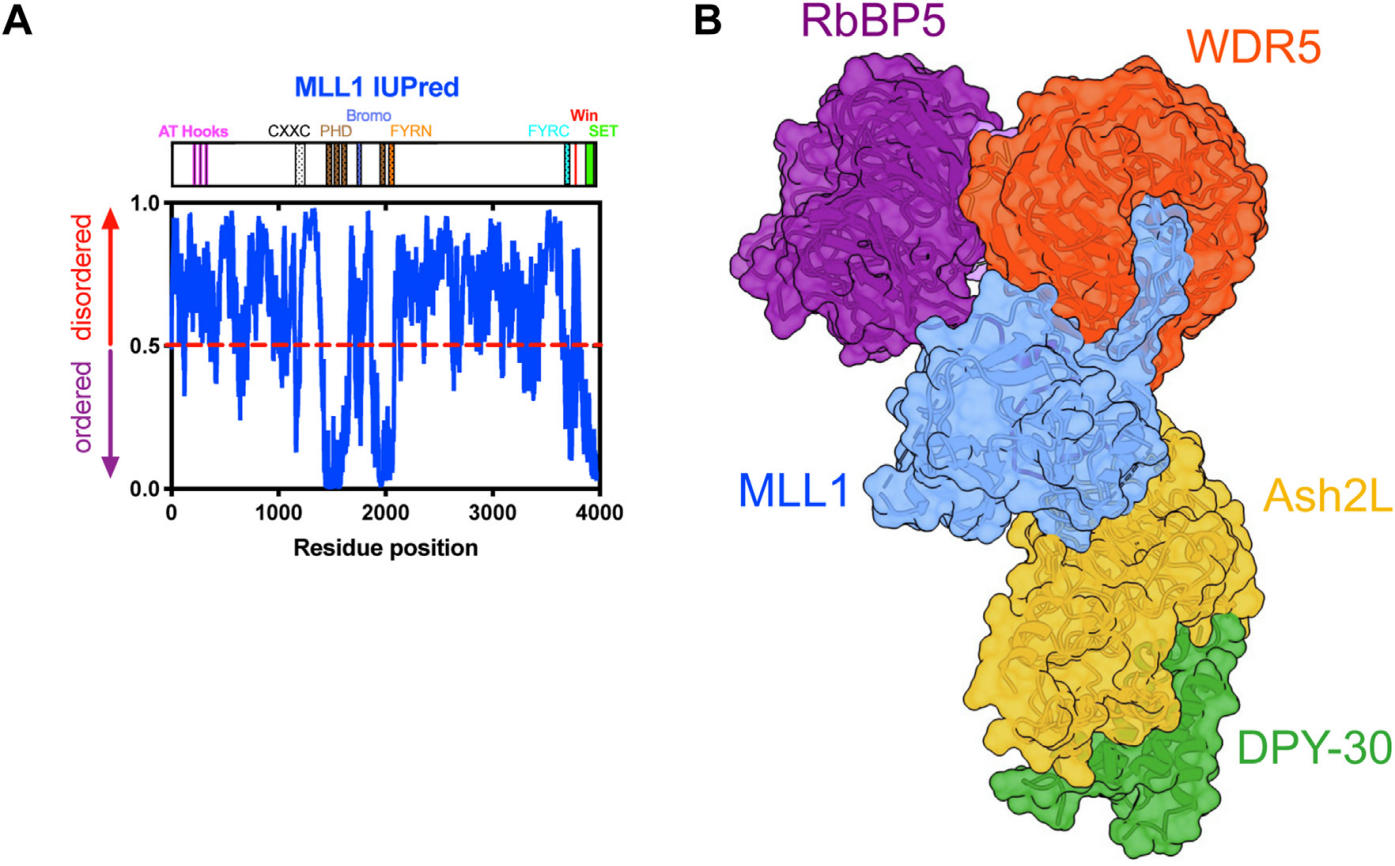
**MLL1 has large regions of intrinsic disorder outside of the SET domain.***A*, IUPRED prediction of the full MLL1 protein (Note: this IUPRED is reproduced in Fig. S1*A*, alongside the IUPRED predictions for the other members of the SET1 family, to showcase their similarities). *B*, structure of the MLL1 core complex (PDB ID: 7UD5). Note: this representative structure is one of the many MWRAD_2_ cryo-EM structures determined on the nucleosome core particle. MLL, Mixed lineage leukemia; SET, Suppressor of Variegation, Enhancer of Zeste, Trithorax.

Biomolecular condensates are membrane-less liquid-like organelles, or intracellular phase-separated compartments, that function to concentrate proteins and nucleic acids to regulate a variety of biological processes (19, 20). This form of compartmentalization has been shown to have variable effects on the activity of enzymes, ranging from a 2- to 70-fold stimulation in the rate of enzyme or ribozyme-catalyzed cleavage reactions to inhibition of catalyzed reactions, protein conformational alterations and increased thermal resistance (21, 22, 23, 24, 25, 26). Whether MLL1 core complex assembly and enzymatic activity is regulated by phase separation is unknown but suggested by multiple primary sequences with regions of low complexity or intrinsically disordered regions (Figs. S1; Tables S6, S2 and S7) that provide numerous transient multivalent interactions involved in liquid-liquid demixing (27) and by the punctate distribution of MLL1 within mammalian cell nuclei (28), a common feature of phase-separated proteins (27).

In this investigation, we combine enzyme kinetics and sedimentation velocity analytical ultracentrifugation with differential interference contrast (DIC) and florescence microscopy techniques to show that phase separation overcomes the thermodynamic barrier for MLL1 core complex assembly and enzymatic activity at physiological temperature. Furthermore, by studying the hydrodynamic changes in the MLL1 core complex, we found that phase separation induces formation of a highly active oligomeric state or scaffold that increases H3K4 mono- and di-methylation activity by ∼35-fold and ∼60-fold, respectively, beyond that expected for a mass action mechanism alone. Our results offer insights into a potential “switch-like” mechanism for spatiotemporal control of H3K4 methylation through the rapid formation or dissolution of biomolecular condensates within eukaryotic transcription factories.

## Results

### Modulation of ionic strength or macromolecular crowding induces MLL1 core complex phase separation

We recently characterized the hydrodynamic and kinetic properties of the catalytic module of the human MLL1 core complex reconstituted with MLL1 residues 3745 to 3969, which is the minimal SET domain fragment required for complex assembly (17, 29) (Fig. 1*B*). We found that the disassembled state of the MLL1 core complex is favored at physiological temperature (37 °C), which results in irreversible enzyme inactivation and low catalytic activity (17). Since increased protein concentration partially restores enzymatic activity, we tested whether the catalytic module of the MLL1 core complex may be regulated by inducing high local concentrations *via* liquid-liquid phase separation (LLPS). We examined several concentrations up to 75 mg/ml of MWRAD_2_ in storage buffer using DIC microscopy but observed no evidence for phase separation. Since alteration of ionic strength is commonly used to induce and study protein LLPS (30), we hypothesized that the lack of MLL1 core complex LLPS may be due to the high ionic strength of the storage buffer, which included 300 mM NaCl. Indeed, a previous investigation showed increased enzymatic activity of the MLL1 core complex with reduced ionic strength (31). We therefore tested whether reduced ionic strength may also regulate the enzymatic activity and the LLPS properties of the MLL1 core complex.

**Figure 2:**
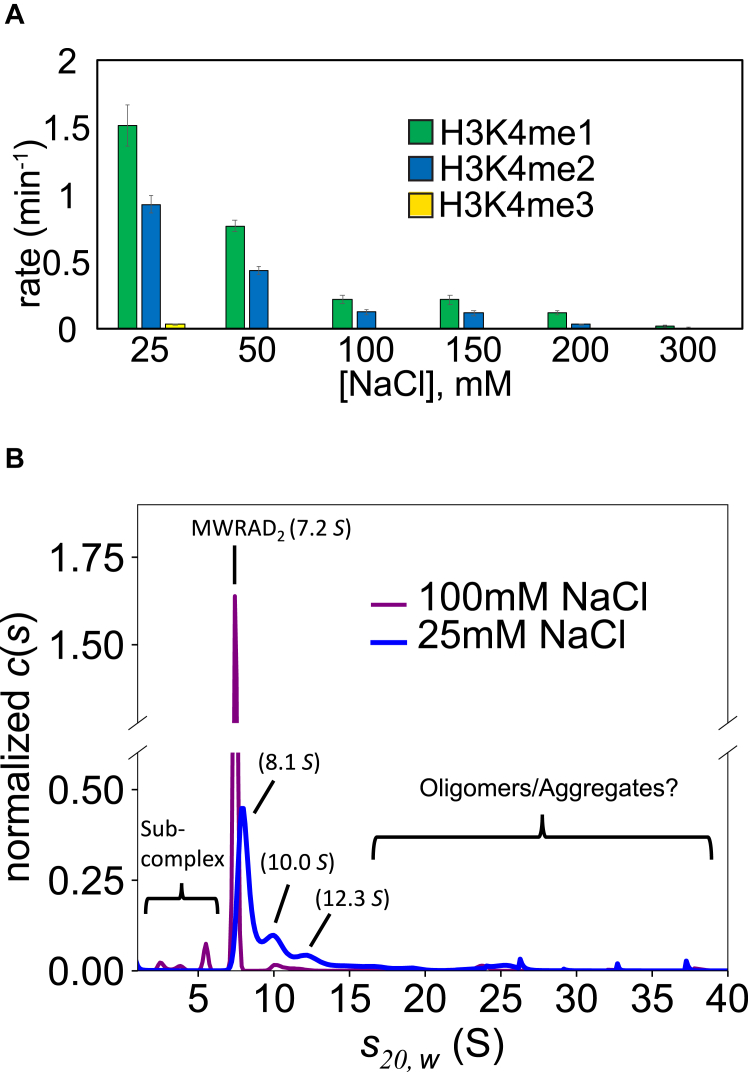
**Reduced ionic strength increases activity and oligomerization of the MLL1 core complex.***A*, comparison of 5 μM MLL1 core complex enzymatic activity at different ionic strengths at 25 °C using the label-free quantitative MALDI TOF mass spectrometry assay. Error bars represent the SD from duplicate measurements. *B*, comparison of 5 μM MLL1 core complex diffusion deconvoluted sedimentation coefficients (c(s)) at 100 mM (*purple line*) and 25 mM (*blue line*) NaCl. The Y-axis was cut between 0.6 and 1.3 to make the larger S-value species more visible. MLL, Mixed lineage leukemia.

First, we compared MLL1 core complex activity at several different ionic strengths at 25 °C using quantitative MALDI-TOF mass spectrometry (15). Consistent with the previous report (31), we found that the enzymatic activity was significantly increased when ionic strength was reduced (Fig. 2*A*). To better understand the reason for increased enzymatic activity, we compared the hydrodynamic properties of the 5 μM MWRAD_2_ complex at moderate (100 mM NaCl) and low (25 mM NaCl) ionic strengths at 25 °C using sedimentation velocity analytical ultracentrifugation (SV-AUC). Comparison of diffusion deconvoluted sedimentation coefficient distributions (*c(s)*) (32) showed unexpected hydrodynamic changes in the complex that were associated with increased enzymatic activity. In contrast to the relatively monodisperse 7.2 *S* peak of the MLL1 core complex at moderate ionic strength (Fig. 2*B*, purple line), the sample became more polydisperse when ionic strength was reduced (Fig. 2*B*, blue line), with peaks at 8.1 *S* (53%), 10.0 *S* (21%), and 12.3 *S* (∼10%), along with several higher molecular weight species that collectively account for ∼16% of the total signal. Furthermore, subcomplex peaks at ∼5 *S* disappeared at low ionic strength (Fig. 2*B*). These results suggest that lower ionic strength not only promotes complex assembly but also induces hydrodynamic alterations in the complex that could include conformational alterations, oligomerization, aggregation, and/or phase separation.

**Figure 3:**
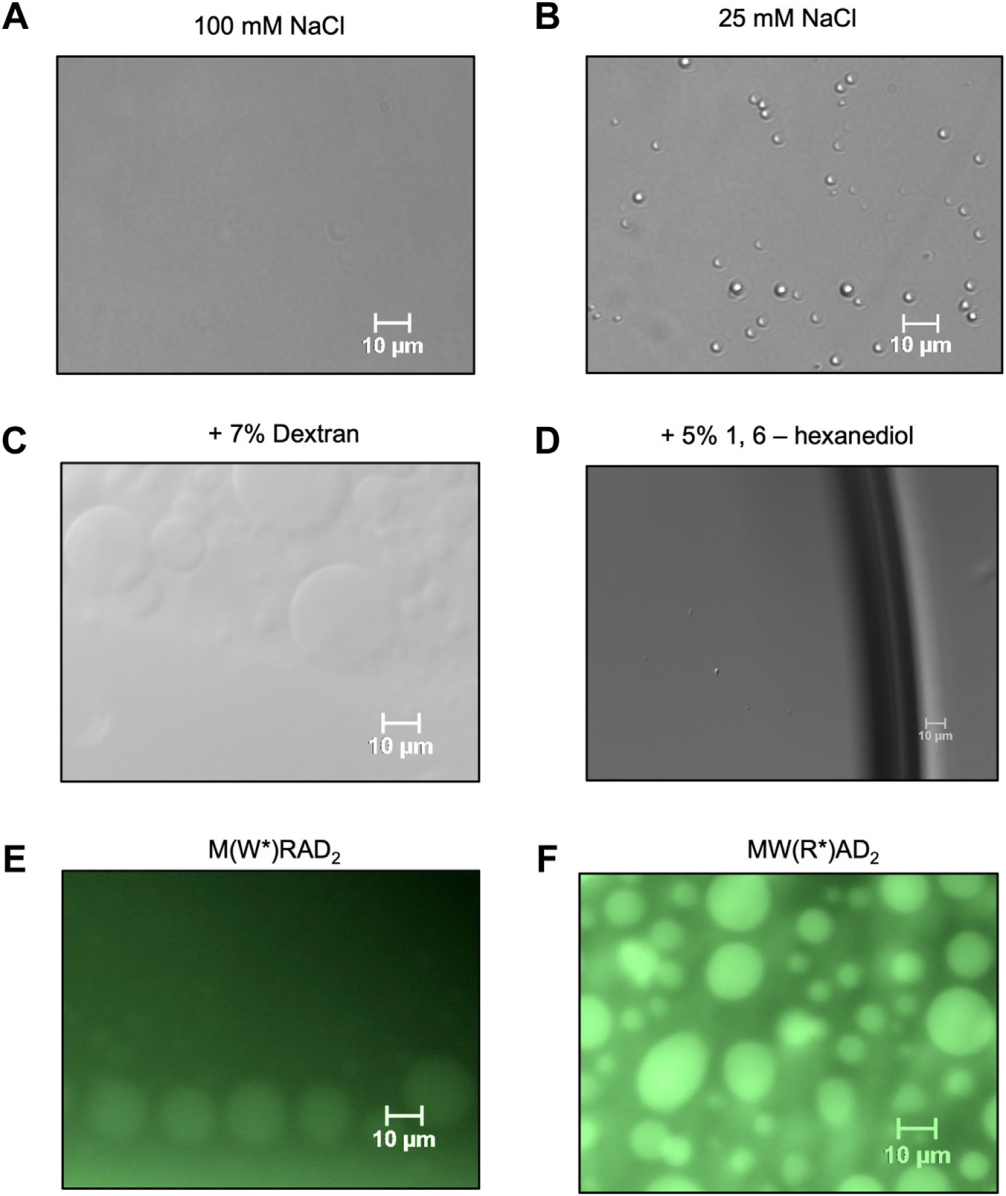
**The MLL1 core complex undergoes LLPS under reaction conditions at low ionic strength or with macromolecular crowding.***A*, DIC microscopy images of MLL1 core complex enzymatic reactions at 100 mM or (*B*) 25 mM NaCl. Each reaction contained 5.0 μM MWRAD_2_, 100 μM H3^1–20^ peptide, and 250 μM SAM in reaction buffer at 25 °C. *C*, the same as in (*B*) but with 7% dextran (see also Movie S1). *D*, same as in (*C*) but with 5% 1,6 hexanediol. *E*, fluorescence microscopy image of the MLL1 core complex assembled with AlexaFluor 488–labeled WDR5 or (*F*) RbBP5 subunits (see Movies S3 and S4). The conditions were 5.0 μM gel filtration–purified complex (see Fig. S3) in reaction buffer with 10 μM H3^1–20^ peptide, 250 μM SAM, 150 mM NaCl, and 7% dextran. DIC, differential interference contrast; LLPS, liquid-liquid phase separation; MLL, Mixed lineage leukemia.

To begin to distinguish among these hypotheses, we compared DIC microscopy images of the enzymatic reaction mixtures. Surprisingly, despite using a relatively low concentration of enzyme (5 μM), the lower ionic strength reaction mixtures showed evidence of spherical LLPS droplets that were absent in the higher ionic strength reaction mixtures (compare Fig. 3, *A* and *B*). The LLPS droplets were small and mobile but did not appear to fuse, which is a common feature of particles induced to undergo LLPS (20). However, addition of a macromolecular crowding agent (dextran; 3–7% w/v) to the reaction mixture resulted in LLPS droplets with larger diameters and observable fusion events that could be detected by DIC microscopy (Fig. 3*C* and Movie S1). Furthermore, in the presence of dextran, the LLPS droplets could be observed at both subphysiological and physiological ionic strengths (Movie S2) and we found that they disappeared in the presence of the LLPS probe 1,6-hexanediol (5%) (Fig. 3*D*), which has been shown to disrupt dynamic liquid-like assemblies, but not protein aggregates (33).

We previously showed that MWRAD_2_ is hierarchically assembled from MW and RAD_2_ subcomplexes (17). To confirm that the droplets contain both subcomplexes, we assembled MWRAD_2_ complexes where representative members of the MW and RAD_2_ subcomplexes (WDR5 and RbBP5) were labeled with FITC, which did not appreciably disrupt assembly or enzymatic activity of the complexes (Fig. S3). Fluorescence microscopy showed signal for both subcomplexes within and outside of the LLPS droplets, suggesting each were in a dynamic equilibrium between compartments (Fig. 3, *E* and *F* and Movies S3 and S4). Together, these results show that the MLL1 core complex can be induced to adopt dynamic LLPS particles by modulation of ionic strength or the macromolecular crowding environment. Furthermore, the sensitivity of the LLPS droplets to aliphatic alcohol 1,6-hexanediol suggests that weak hydrophobic protein–protein interactions are involved.

### WDR5, RbBP5, and Ash2L subunits may drive MLL1 core complex phase separation

While full-length MLL1 contains large intrinsically disordered regions that may drive phase separation (Fig. 1*A*), the MLL1 construct used in this investigation contains comparatively few regions of predicted disorder (Fig. S2*A*), suggesting LLPS of the catalytic SET domain may be driven by other subunits of the complex having variable degrees of predicted disorder (Fig. S2, *B*–*E*). Indeed, use of the PScore (34) and CatGRANULE (35) phase separation prediction programs suggests that the MLL1 construct used in this investigation has a low phase separation probability (Table S1). In contrast, Ash2L and Ash2L-containing complexes show high LLPS probability scores (Table S1), suggesting that it may drive MLL1 core complex phase separation. The recent cryo-EM structures of the MLL1 core-complex and several others (36, 37, 38) demonstrate that some intrinsically disordered regions of MWRAD_2_ become more constrained in the complex and/or upon interaction with the nucleosome core particle. For instance, the unstructured “hinge” region of RbBP5 is more-or-less constrained through multiple interactions with MLL1, WDR5, and Ash2L, providing it with enough order to be visible in cryo-EM models. However, it also appears that multiple intrinsically disordered regions from WDR5 (aa. 1–42), RbBP5 (aa. 370–538), Ash2L (aa. 1–274), and DPY30 (aa. 1–44) are still sufficiently flexible to provide poor electron density, resulting in their omission from the final structural model. Many of these tails are likely available in the complex to form the contacts required to drive LLPS. Importantly, this structural observation alone does not define which of the tails are necessary to the stability of the complex or serve as drivers of the LLPS mechanism.

To determine which subunit(s) may drive MLL1 core complex LLPS, we compared DIC microscopy images of the individual subunits under similar conditions. Consistent with the predictions from the PScore and CatGRANULE programs, LLPS droplets were observed for Ash2L and not for MLL1 and DPY-30 subunits (Fig. S4). Surprisingly, despite low LLPS probability scores, both WDR5 and RbBP5 were observed to undergo LLPS in isolation from the other MWRAD_2_ components (Fig. S4). These results suggest that MLL1 core complex phase separation may be driven by WDR5, RbBP5, and/or Ash2L subunits.

### Phase separation stimulates MLL1 core complex enzymatic activity

**Figure 4:**
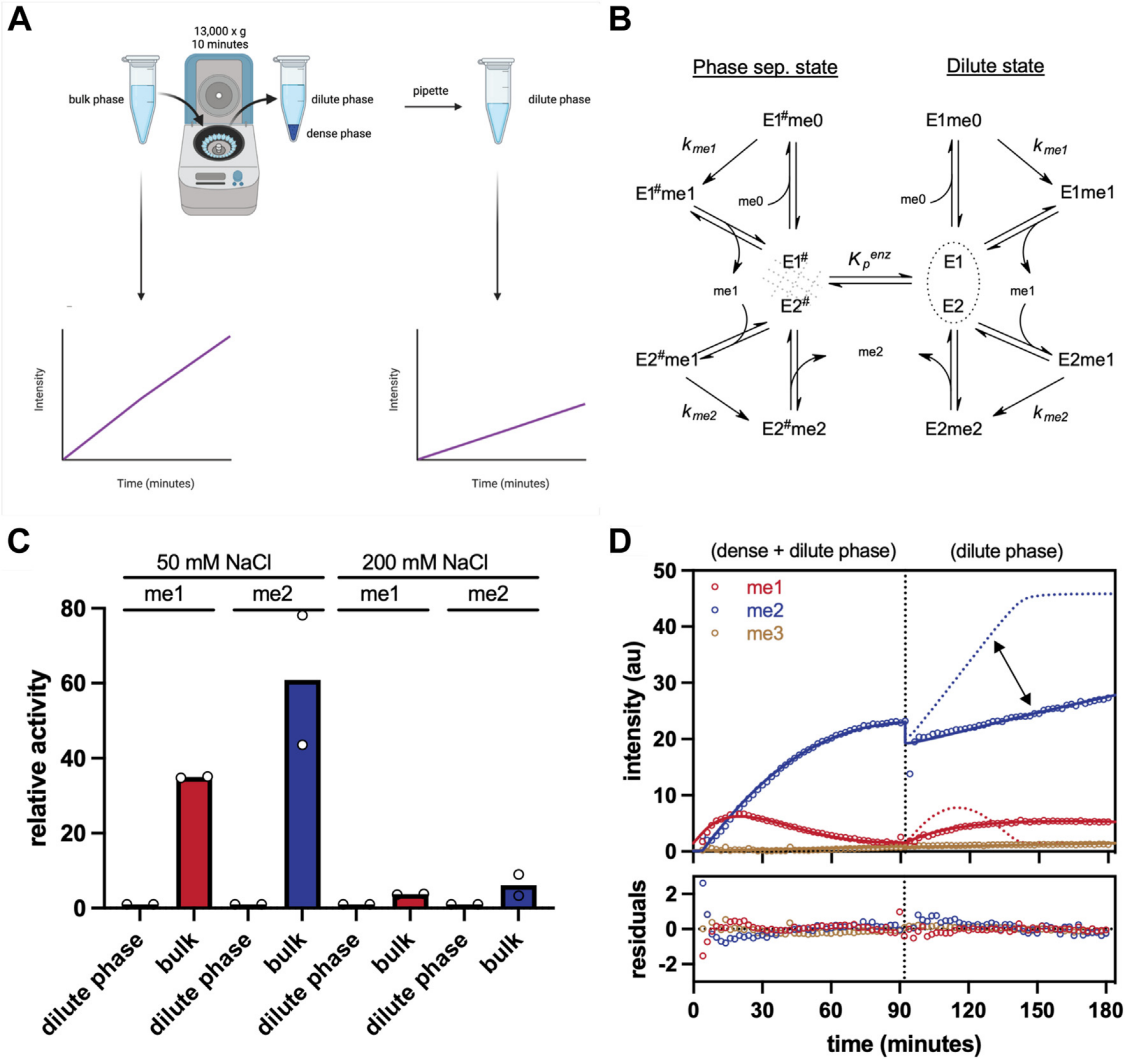
**A condensate activity assay demonstrates the difference in activity between the “dense” and “dilute” phases of MWRAD**_**2**_**.***A*, schematic of the condensate activity assay, showing the anticipated decrease in rate between the “bulk” phase and the “dilute” phase. Figure adapted from Tibble *et al.* (41). Schematic made with Biorender.com. *B*, minimal kinetic model for MWRAD_2_ complex formation and activity in both the phase-separated state (*left* side) and dilute state (*right* side). (me0 – unmethylated H3 peptide, me1 – monomethyl H3K4 peptide, me2-dimethyl H3K4 peptide, *K*_*p*_^*enz*^ - enzymatic partition coefficient, *k*_*me1*_ and *k*_*me2*_ are the pseudo first-order rate constants (*k*_*cat*_) for the mono- and di-methylation reactions, respectively.) *C*, histogram showing the difference in relative activity between the dilute phase and bulk phase of each reaction for both 50 mM and 200 mM NaCl. *D*, a time course of the 50 mM NaCl reaction from 0 min to 90 min, followed by centrifugation and incubation with additional H3 peptide and SAM for an additional 90 min. The *dotted lines* represent the amount of expected mono- (*red*) and di-methylation (*blue*) activity if there was no depletion of enzyme after centrifugation. H3K4, histone H3 lysine 4.

To determine if the increased enzymatic activity is inside or outside of the droplets, we performed single turnover condensate activity assays using real time heteronuclear NMR spectroscopy (Fig. 4*A*) (39). Since each methylation state exhibits unique chemical shifts in ^1^H-^13^C-HSQC experiments (Fig. S5*A*), we measured H3K4me1, me2, and me3 intensities over time and fit the data using Kintek explorer software (https://kintekcorp.com/software) (40) (Fig. 4, *B*–*D*). First, we measured activity in a bulk phase mixture, containing the MLL1 core complex inside (dense phase) and outside (dilute phase) of the LLPS droplets, after addition of histone H3 peptide and ^13^C-methyl-labeled SAM (^13^C-SAM). We then separated the dense and dilute phases by centrifugation (41), followed by addition of unmethylated histone H3 peptide and ^13^C-SAM to the dilute phase and measured product formation (dilute phase methylation). Because the droplets were too small for direct measurements, the differences in the rate of methylation between the two samples were attributed to enzyme sequestered in the droplets.

The data for both bulk phase and dilute phase experiments were globally fit using a simulation we developed in Kintek explorer software (Fig. 4*B*). This simulation assumes that the activity in the bulk phase samples derives from the sum of activities from two states of the enzyme, the phase-separated state (E1^#^E2^#^) and dilute state (E1E2), the equilibrium of which is described by a fitted value we called the enzymatic partition coefficient (*K*_*p*_^*enz*^) (Fig. 4*B*). The reaction in each state is modeled assuming a nonprocessive reaction in which the monomethyl product is released from the E1 active site, which then binds to the second active site (E2) within the complex for the di-methylation reaction, as described previously (17). Turnover numbers (*k*_*cat*_) for the mono- and di-methylation reactions were assumed to be identical for each state of the enzyme and were fixed, allowing estimation of the catalytic efficiency (*k*_*cat*_*/K*_*m*_) for each reaction. Negligible H3K4 trimethylation was observed under these conditions and did not produce reliable estimates of kinetic parameters.

Using this simulation, we compared bulk and dilute phase methyltransferase activities under phase separation (50 mM NaCl) and nonphase separation (200 mM NaCl) conditions. When assayed under phase separation conditions, H3K4 mono- and di-methylation activities were ∼35-fold and ∼60-fold greater, respectively, in the bulk sample than the dilute phase (Fig. 4, *C* and *D*). In contrast, relatively small differences in enzymatic activity (3–6 fold) were observed between bulk and dilute phase samples when the assays were conducted in 200 mM NaCl (Figs. 4*C* and S5*B*). We conclude from these experiments that the increased activity is occurring within the LLPS droplets. Furthermore, phase separation appears to have a greater impact on the H3K4 di-methylation reaction.

The fitted enzymatic partition coefficient (*K*_*p*_^*enz*^) under nonphase separation conditions (200 mM NaCl) was 0.23, suggesting that in the dilute phase, the nonoligomeric state of the enzyme was favored. In contrast, under phase separation conditions, *K*_*p*_^*enz*^ was 1.4, suggesting a ∼six-fold enrichment of enzyme in the dense state. Consistent with a small population of an active oligomeric species, and the small total volume of the dense phase seen in DIC images, SDS gels of the postreaction samples indicate that a modest fraction of the total available MLL1 core complex was found in the dense phase (Fig. S6), and yet the enzymatic activity is largely associated with this phase. This result indicates that the 30- to 60-fold increase in activity under phase separation conditions cannot be explained solely by a mass action mechanism that increases local concentration of enzyme and substrate within a droplet. Instead, the altered hydrodynamic properties observed in the SV-AUC experiments suggests that phase separation induces formation of a small population of a unique and highly active oligomeric species that is not present to the same extent under nonphase separation conditions.

A similar droplet pelleting approach has become standard in the field and was required to examine activity differences between phases by Peeples and Rosen (42) who suggested that enzymatic reactions in condensates are accelerated by contributions from both mass action and changes in substrate *K*_*m*_ induced by a molecular reorganization of the enzyme into a scaffold. Since the SV-AUC experiments above showed evidence of higher molecular weight species that could be evidence of such a scaffold, we compared apparent *K*_*m*_ values between the bulk and dilute phase samples. Under phase-separated conditions, the apparent *K*_*m*_ for unmethylated histone H3 (H3K4me0) went from 2300 μM in the dilute phase to 65 μM in the bulk sample (Table S2). Likewise, the apparent *K*_*m*_ for the H3K4me1 substrate in the di-methylation reaction went from 163 μM in the dilute phase to ∼8 μM in the bulk sample (Table S2). Together, these results are consistent with a scaffold-induced decrease in substrate *K*_*m*_.

### Conformational and quaternary structural changes are associated with MLL1 core complex phase separation

**Figure 5:**
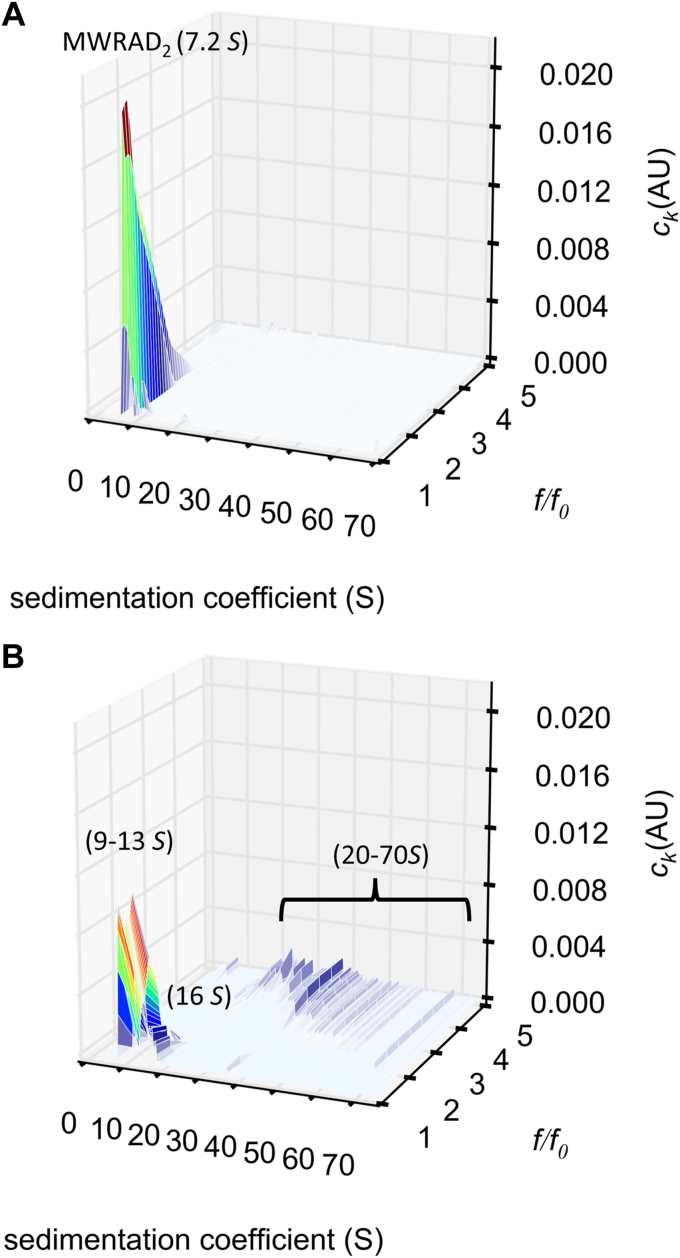
**Changes in [NaCl] result in altered hydrodynamic properties of MWRAD**_**2**_**.***A*, size and shape analysis (*c(s,f*_*r*_*)*) of 5 μM MWRAD_2_ in 100 mM NaCl buffer. *B*, same as (*A*) but with 25 mM NaCl buffer.

LLPS is often associated with unusual hydrodynamic alterations of proteins and protein complexes (43, 44). In the case of the MLL1 core complex, these alterations could be quantified by SV-AUC. To further characterize this unusual hydrodynamic behavior, we performed a two-dimensional size and shape distribution analysis *(c(s,f*_*r*_*))* of the SV-AUC data from Figure 2*B*, which allows estimation of the frictional coefficients and average molar masses of each species in a complex distribution (45). This two-dimensional analysis capability provides a uniquely powerful biophysical method for analyzing polydisperse systems (46). The *c(s,f*_*r*_*)* distribution of MWRAD_2_ at moderate ionic strength showed a single peak with the typical experimental *S*-value of the complex but encompassing a broad range of frictional ratios between 1 and 3, with a weight-average frictional coefficient of ∼1.5 (Fig. 5*A*). The estimated average molecular mass using this frictional coefficient and *S-*value was ∼190 kDa, which is less than 10% error of the theoretical mass of the monomeric complex (205 kDa). In contrast, under phase separation conditions, the *c(s,f*_*r*_*)* distribution showed that the majority of the signal is divided among several peaks with larger *S-*values ranging between 8 and 16, with evidence of numerous larger molecular weight species between 20 to 70 *S* (Fig. 5*B*). Several of the peaks between 8 and 13 *S* had frictional ratios that ranged between 1.1 and 1.2, which gave mass estimates between 140 and 230 kDa. Because these species have relatively similar molar mass estimates, these *S*-values likely correspond to species with increasingly compact conformations of the monomeric MLL1 core complex that allow them to sediment faster. The peak at ∼16 *S* gave a mass estimate of ∼350 kDa, which is indicative of a reaction boundary between monomeric and dimeric complexes. These results suggest that lower ionic strength allows the complex to sample different conformational states, some of which are more compact and some that allow oligomerization of the MLL1 core complex. Consistent with this interpretation, these larger *S*-value species become increasingly more populated in an MWRAD_2_ concentration-dependent manner (Fig. S7). The larger *S*-value species between 20 to 70 *S* showed a broader range of frictional ratios ranging between 3 to 5 (Fig. 5*B*). Integration of these peaks gave mass estimates starting at ∼3.7 MDa, which approximates an 18-mer of MWRAD_2_, with each discrete species at higher *S*-values approximating the addition of one MWRAD_2_ dimer. This hydrodynamic behavior is indicative of fiber-like scaffold particles (46) and likely reflects the pre-initiation intermediates associated with the formation of phase-separated droplets (20, 27). These results reveal that phase separation is associated with dynamic conformational and quaternary structural changes of the MLL1 core complex.

### Increased activity is associated with the formation of a highly active oligomeric scaffold of the MLL1 core complex

**Figure 6:**
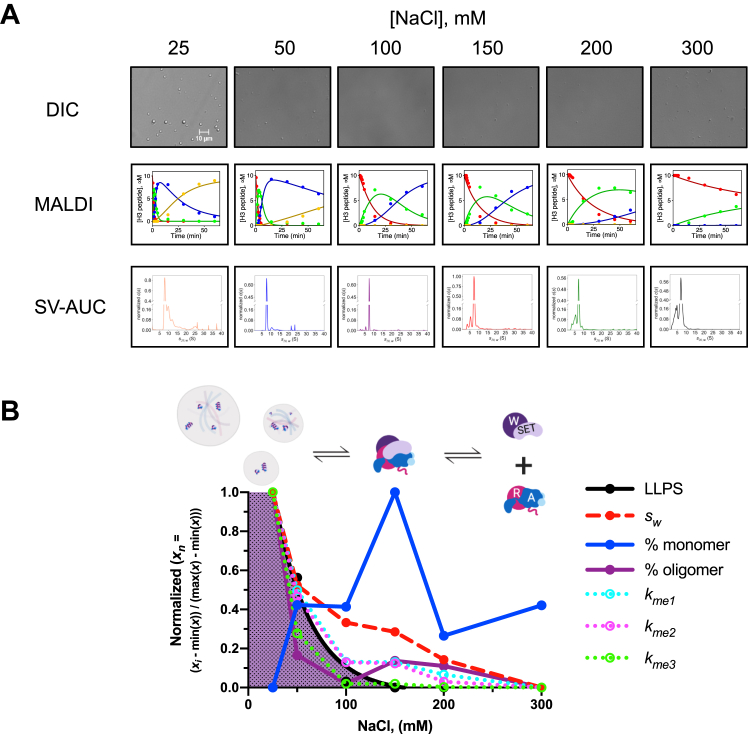
**Phase separation–induced hydrodynamic alterations of the MLL1 core complex are directly related to increased enzymatic activity.***A*, 5 μM MWRAD_2_ was mixed with 100 μM histone H3 peptide and 250 μM SAM at the indicated NaCl concentrations at 25 °C. DIC microscopy images (*top row* – note: the 25 mM NaCl image (*far left*) is reproduced from Figure 3*B*, as they are the same information, presented here in the context of the other NaCl concentrations assayed for comparison), enzymatic reaction progress curves (*middle row*), and SV-AUC c(s) plots (*bottom row*) are displayed for each reaction mixture. Peptide species in the reaction progress curves were as follows: H3K4me0 (*red*), H3K4me1 (*green*), H3K4me2 (*blue*), and H3K4me3 (*yellow*). *B*, summary of biophysical parameters as a function of NaCl concentration. Each parameter was min-max normalized as indicated on the ordinate axis to place them on the same scale. The best fit of the LLPS score was obtained with a one phase exponential decay equation using Prism version 9 (R squared = 0.99) (*solid black line*). The *purple shaded* area shows conditions where LLPS was observed. Pseudo first-order rate constants (*k*_*me1,2,3*_) at each NaCl concentration are shown with the *dotted lines*. Signal weight-average sedimentation coefficient (*s*_*w*_) derived from integration of each c(s) profile is shown with the *red dashed line*. Percent monomer and oligomer are shown with the *solid blue* and *purple* lines, respectively. A schematic representing each biophysical state is shown above the plot. (Created with BioRender.com). H3K4, histone H3 lysine 4; DIC, differential interference contrast; LLPS, liquid-liquid phase separation; MLL, Mixed lineage leukemia; SV-AUC, sedimentation velocity analytical ultracentrifugation.

While it is generally agreed that increased concentration of enzyme and substrate within a droplet stimulates enzymatic activity *via* a mass action mechanism, additional mechanisms merit exploration for the MLL1 core complex. Specifically, we have previously shown that the MLL1 core complex occupies a disassembled state at physiological temperatures and concentrations that disfavor phase separation, which in turn dramatically reduces the H3K4 methylation activity (17). These findings raise the question of how much the increased enzymatic activity under phase separation conditions is due to the altered hydrodynamic state of the MLL1 core complex and to what extent they are independent of mass action and ionic strength effects on reaction chemistry. If the changes in enzymatic activity were purely due to ionic strength effects on the reaction, then we would expect to observe a direct linear relationship between ionic strength and enzymatic activity when the complex was assayed under conditions where it is mostly assembled. In contrast, if the changes in methylation rate were not salt-dependent, then we would expect to observe a nonlinear relationship in methylation rate as a function of salt concentration. To test this prediction, we compared DIC microscopy images, enzymatic activity, and SV-AUC profiles at several NaCl concentrations using reaction mixtures where the MLL1 core complex was at a concentration and temperature (5 μM at 25 °C)—that was well above the *K*_*d*_^*app*^ for complex assembly (62 nM (17)) (Figs. 6*A* and S8). We then compiled a table of calculated and experimental parameters that define the enzymatic and hydrodynamic properties of the MLL1 core complex, including ionic strength, LLPS score, overall signal-weighted average *S*-value (*s*_*w*_), and relative amounts of integrated signal corresponding to the MWRAD_2_ monomer and oligomer in SV-AUC experiments (Table 1). Min-Max normalization of the results from each experiment allowed us to compare variability of the respective parameters at different ionic strengths (Fig. 6*B*). The resulting phase diagram reveals that the MLL1 core complex began phase separating near physiological ionic strength (150 mM NaCl) and increased exponentially when ionic strength was further reduced (Fig. 6*B*). Contrary to our prediction, we did not observe a direct linear relationship between ionic strength and enzymatic activity. Instead, variability in methylation rates mirrored the LLPS score, which showed strong linear associations with each methylation event (Pearson’s correlation r = 0.96–0.99 *p* ≤ 0.002, Fig. S9*A* and Table 1). Changes in enzymatic activity were also strongly correlated with the altered hydrodynamic properties of the MLL1 core complex as reflected by changes in the *s*_*w*_ value (Pearson’s correlation r = 0.94–0.98 *p* ≤ 0.005, Fig. S9*B*) and the relative amount of oligomeric MLL1 core complex (Pearson’s correlation r = 0.94–0.98 *p* ≤ 0.007, Fig. S9*C*). In contrast, the more modest inverse correlations between enzymatic activity and ionic strength or % monomer did not rise to the level of statistical significance (*p* = 0.06–0.14 *k*_*me1*_–*k*_*me3*_, Fig. S9, *D* and *E*, respectively). Indeed, a nonrandom distribution of residuals was evident when ionic strength was regressed on each methylation event (Fig. S9*D*), indicating that the LLPS-induced variation in enzymatic activity is likely not directly due to independent ionic strength effects on reaction chemistry.

**Table 1:** Correlation matrix (Pearson)^a^

Variables	*k*_*me1*_	*k*_*me2*_	*k*_*me3*_	Ionic strength	*LLPS score*	*s*_*w*_	% Monomer	% Oligomer
*k*_*me1*_	**1**	**0.999**	**0.978**	−0.795	**0.989**	**0.977**	−0.567	**0.931**
*k*_*me2*_	**0.999**	**1**	**0.982**	−0.787	**0.985**	**0.978**	−0.561	**0.937**
*k*_*me3*_	**0.978**	**0.982**	**1**	−0.676	**0.961**	**0.940**	−0.629	**0.977**
Ionic strength	−0.795	−0.787	−0.676	**1**	−0.754	**−0.883**	0.269	−0.616
*LLPS score*	**0.989**	**0.985**	**0.961**	−0.754	**1**	**0.940**	−0.606	**0.891**
*s*_*w*_	**0.977**	**0.978**	**0.940**	**−0.883**	**0.940**	**1**	−0.484	**0.906**
% monomer	−0.567	−0.561	−0.629	0.269	−0.606	−0.484	**1**	−0.578
% oligomer	**0.931**	**0.937**	**0.977**	−0.616	**0.891**	**0.906**	−0.578	**1**

a Values in bold are different from zero with significance level α = 0.05.

**Figure 7:**
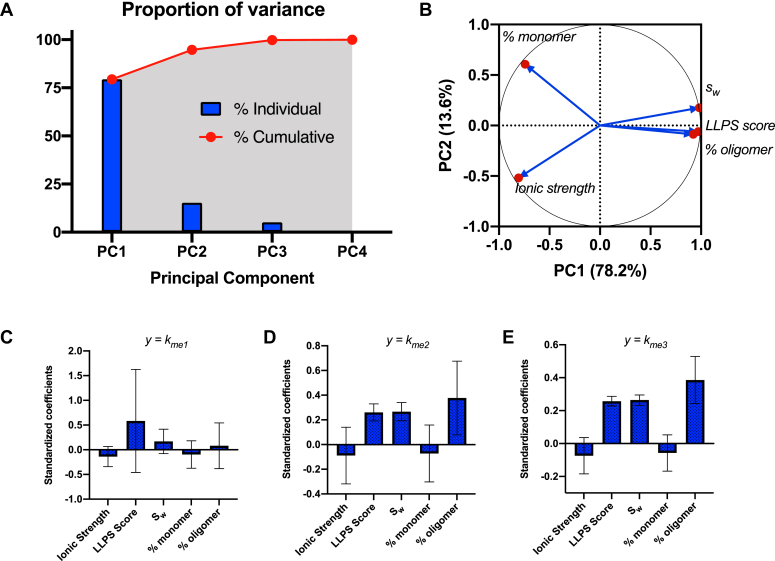
**Principal component regression analysis of biophysical parameters impacting variability in MLL1 core complex H3K4 methylation activity.***A*, proportion of variance plot showing the relative contributions of each principal component (*blue bars*), as well as cumulative contributions (*red points* and *shaded* area), to the overall variation. *B*, PCA loading plot showing the relationships among the variables. *LLPS* score and hydrodynamic parameters (*s*_*w*_ and *%* oligomer) group together indicating that they are correlated and strongly influence *PC1.* % monomer and ionic strength are the strongest contributors to PC2. *C*–*E*, PCR standardize regression coefficients for each parameter obtained from the regression of principle components accounting for at least 80% of the variability on rates of H3K4 mono- (*C*), di-methylation (*D*), and trimethylation (*E*). Error bars represent the 95% confidence interval. H3K4, histone H3 lysine 4; LLPS, liquid-liquid phase separation; MLL, Mixed lineage leukemia; PCA, principal component analysis; PCR, principal component regression.

To evaluate the contribution of each of these parameters to the variation in enzymatic activity, we performed principal component regression (PCR) analysis. PCR is a statistical approach that overcomes the limitations of collinearity among independent variables in a multiple regression analysis by grouping sets of covarying explanatory variables into uncorrelated new variables, called principal components, that can then be used in a regression analysis to examine how each parameter contributes to the variability in the H3K4 methylation rates (see Experimental procedures). The initial principal component analysis revealed that 92.8% of the overall variability in the data is described by two principal components (Fig. 7*A* and Table S3). The third and fourth principal components only accounted for 5.9% and 1.4% of the overall variability in the data. The variables representing LLPS-induced formation of the oligomeric scaffold (*s*_*w*_, % oligomer, and LLPS score) contributed most strongly and equally to principal component 1 (Fig. 7*B* and Table S3), which accounted for 79.2% of the observed data variability and were the only individual parameters to demonstrate statistically significant relationships with the rates of H3K4 methylation. The variables representing contributions of mass action (% monomer) and ionic strength contributed most strongly to principal component 2, which accounted for 13.6% of the overall data variability (Fig, 7*A* and Table S3).

Given the strong contribution from LLPS-induced formation of the oligomeric scaffold as a driver of the variation in enzymatic activity in our data sets, we next sought to establish which variables exert the greatest mechanistic control over monomethylation, in contrast to di-methylation or trimethylation. Linear regression of the H3K4 methylation rate vectors on the principal components revealed statistically significant models for each methylation reaction (ANOVA *p* ≤ 0.01), with principal component 1 being the most important predictor for each. Transformation of the resulting regression vectors back to the scale of the input parameters revealed the relative contributions of each parameter to the variability in H3K4 methylation rates (Fig. 7, *C* and *D*). Interestingly, when the rate of H3K4 monomethylation was the dependent variable, variability of the LLPS score appeared to be the largest contributor to the variance, with relatively minor contributions from ionic strength and the hydrodynamic parameters *s*_*w*_ and % oligomer (Fig. 7*C*). However, the 95% confidence interval for each variable was too wide to reject the null hypothesis. In contrast, when the rates of H3K4 di-methylation or trimethylation were the dependent variables, LLPS score, *s*_*w*_, and % oligomer all displayed statistically significant contributions to the variability in the H3K4 methylation rates, whereas ionic strength and % monomer showed relatively minor contributions with regression coefficients that were not statistically significant (Fig. 7, *D* and *E*). Thus, our PCR analysis suggests that changes in oligomerization drive not only enhancement of MLL1 core complex catalytic rates but also its status as a mono- or di-methyltransferase.

To summarize the findings from this analysis, the increased rates of H3K4 di-methylation under phase separation conditions appear to be driven primarily by the hydrodynamic changes induced by MLL1 core complex oligomerization rather than by independent ionic strength effects on reaction chemistry. Similarly, while variation in ionic strength does not directly explain variability in the rates of H3K4 monomethylation, the monomethylation rate appears to be less dependent on phase separation–induced oligomerization of the MLL1 core complex compared to that of the di-methylation and trimethylation reactions. This result is consistent with the condensate activity assays showing that phase separation has a greater impact on the H3K4 di-methylation reaction (Fig. 4*C*). Together, these results suggest that the conformational changes associated with oligomerization of the MLL1 core complex are required for optimal formation of the di-methylation and trimethylation active site(s), which, under nonphase separation conditions, limits the overall rate of H3K4 methylation.

### Phase separation overcomes thermodynamic barrier for complex assembly and enzymatic activity at physiological temperature

**Figure 8:**
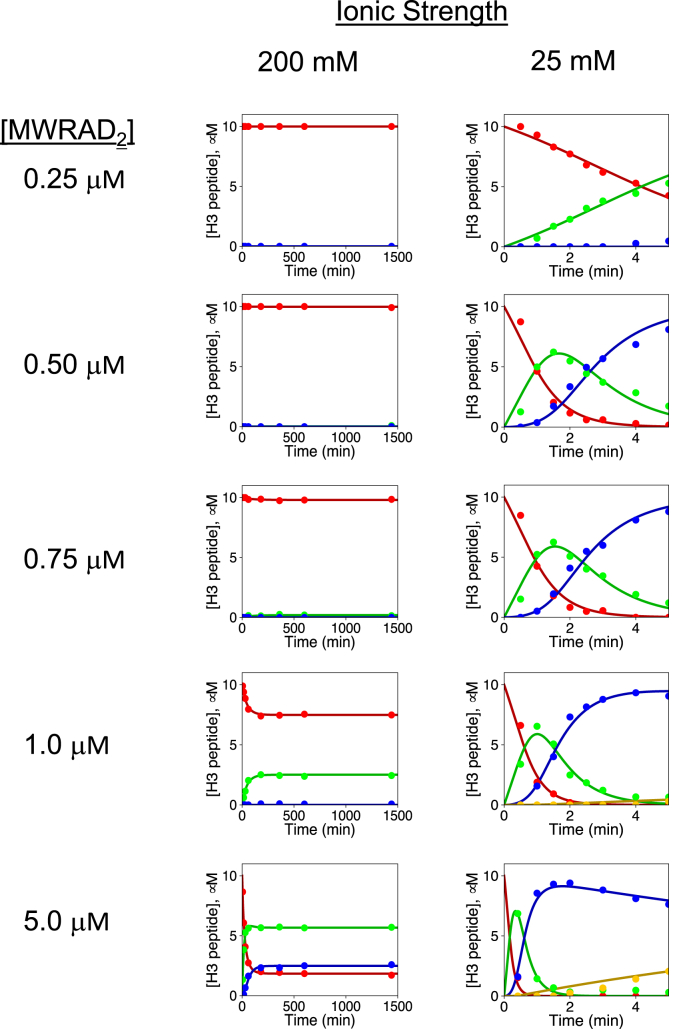
**Enzymatic activity of the MLL1 core complex at physiological temperature under phase and nonphase separation conditions.** Comparison of MLL1 core complex enzymatic activity at the indicated concentrations at 37 °C in high (200 mM NaCl) *versus* low (25 mM NaCl) ionic strength reaction buffers. The 200 mM NaCl panels (*left*) are reused from reference (17) (Fig. 7) for the purpose of comparison. Each time point represents the mean concentration of each peptide species, and *solid lines* show the fit using a modified form of Model 3 from (17) to account for trimethylation. The resulting pseudo first-order rate constants are summarized in Table 2. Peptide species were H3K4me0 (*red*), H3K4me1 (*green*), H3K4me2 (*blue*), and H3K4me3 (*yellow*). Note the time scale differences required for the high *versus* low ionic strength reactions. H3K4, histone H3 lysine 4; MLL, Mixed lineage leukemia.

Lastly, to determine if LLPS formation rescues enzymatic activity at physiological temperature, we compared methylation kinetics of different concentrations of the MLL1 core complex among high or low ionic strength reaction mixtures at 37 °C. At near physiological ionic strength, none of the reactions went to completion, even after 24-h incubation, mainly due to rapid enzyme inactivation at 37 °C (Fig. 8, left column (plots re-used from (17) for comparison)). In contrast, in low ionic strength buffer, most of the tested concentrations showed at least 80% conversion to the di-methylated form of H3K4 after only 5 min (Fig. 8, right column). At the highest concentrations tested (5 μM), the pseudo first-order rate constants for mono- and di-methylation increased 62- and 50-fold, respectively, with no evidence of enzyme inactivation (Table 2). All together, these results are consistent with the hypothesis that LLPS overcomes the thermodynamic barrier for MLL1 core complex assembly and enzymatic activity at physiological temperatures.

**Table 2:** Summary of rate constants at 37 °C in high (200 mM) and low (25 mM) NaCl reaction buffer^a^

[MWRAD_2_], μM	*k*_*1*_ (min^−1^) [NaCl], mM		*k*_*2*_ (min^−1^) [NaCl], mM		*k*_*3*_ (min^−1^) [NaCl], mM		*k*_*inact*_ (min^−1^) [NaCl], mM	
	200	25	200	25	200	25	200	25
0.25	N/A^b^	0.3 (0.01)	N/A	N/A	N/A	N/A	>2000^c^	0.00 (N.D.)
0.5	N/A	1.4 (0.10)	N/A	1.1 (0.20)	N/A	N/A	>390^c^	0.00 (N.D.)
0.75	0.00 (0.02)^d^	1.5 (0.10)	N/A	1.2 (0.09)	N/A	N/A	0.13 (1.00)	0.00 (N.D.)
1.0	0.07 (N.D.)^e^	2.3 (0.20)	N/A	1.9 (0.10)	N/A	0.01 (0.01)	0.24 (0.11)	0.00 (N.D.)
5.0	0.13 (0.06)	8.1 (1.80)	0.09 (0.06)	4.5 (0.30)	N/A	0.03 (N.D.)	0.21 (0.13)	0.00 (N.D.)

a Each is the rate constant +/− (standard error estimate, 95% confidence interval) derived from nonlinear regression fitting of the data to Model 3 from (17). Duplicate measurements were made for each time point.

b N/A, Not applicable – no methylation observed.

c *k*_inact_ lower bound. In Kintek Explorer software, *k*_*me1*_ was fixed to the value predicted by the Arrhenius equation at the indicated temperature and *k*_inact_ was floated to estimate the lower bound required for the observed loss of activity.

d Rates below 5 × 10^−3^ were rounded to 0.00.

e N.D., error estimates are not defined.

## Discussion

We previously found that the MW and RAD_2_ subcomplexes interact with a *K*_*d*_^*app*^ of ∼6 μM at 37 °C (17), raising the question of how complex forms in cells that contain relatively few molecules of MLL1: estimated to be ∼1 fmol per mg of nuclear extract (47). WRAD_2_ subunits appear to be present in cells in vast excess compared to MLL1 (47), which could help overcome the thermodynamic barrier to complex assembly. Alternative possibilities to overcome this barrier in cells include interaction with other proteins, cofactors, nucleic acids; the addition of posttranslational modifications and/or by inducing a high local concentration of MWRAD_2_ subunits within a phase-separated compartment. While there is evidence that phosphorylation, long noncoding RNAs and chaperones regulate the function of MLL family complexes (48, 49, 50), it remains to be determined if these mechanisms would overcome the barrier to MLL1 core complex assembly at physiological temperatures.

Our data suggest that concentration of the MLL1 core complex in a biomolecular condensate overcomes the barrier to complex assembly at physiological temperatures, resulting in histone methyltransferase activity that is increased by at least 30- to 60-fold (Table 2). However, while complex formation is a prerequisite for enzymatic activity, it does not by itself explain the dramatic increase in activity under phase separation conditions. Instead, our results suggest that phase separation is associated with conformational changes that promote a highly active oligomeric scaffold of the MLL1 core complex that reduces substrate *K*_*m*_. This conclusion is supported by the hydrodynamic changes in the complex observed in SV-AUC experiments with reduced ionic strength or simulated macromolecular crowding, both of which showed changes from a relatively monodisperse holo-MLL1 core complex to a polydisperse array of species with masses ranging from that of a compact monomer up to megadalton oligomeric fibers. Similar hydrodynamic changes have been observed in other proteins regulated by phase separation, including the heterochromatin protein HP1α (44), the proteasomal shuttle factor UBQLN2 (43), nucleophosmin (NPM1) (51), and a prion-like domain (52). Based on these observations, we suggest that these SV-AUC–observed hydrodynamic changes represent an important “phase separation signature” that can be used to identify conditions and, as we demonstrate in this investigation, be used to quantify associated tertiary and quaternary structural changes. This information has allowed us to show that the LLPS-induced increase in enzymatic activity of the MLL1 core complex is highly correlated with alterations in the quaternary structure of the enzyme complex.

We also introduce in this investigation the enzymatic partition coefficient (*K*_*p*_^*enz*^). Unlike the traditional definition of the partition coefficient (*K*_*p*_), defined as the ratio of concentrations of protein in the dense and dilute phases, *K*_*p*_^*enz*^ reflects the ratio of protein in the high *versus* low enzymatic activity states. We suggest that both values can be useful in deciphering thermodynamic mechanisms of LLPS-induced activity stimulation. For example, if activity stimulation is due solely to mass action, then we expect the LLPS-induced change in *K*_*p*_ to equal the change in *K*_*p*_^*enz*^. Deviations between the two values, as observed in this investigation, reflect differences in the inherent enzymatic activity between the two states of the enzyme that cannot be explained by mass action alone.

These observations raise the question of how LLPS increases the enzymatic activity of the MLL1 core complex. Our data suggest that LLPS induced rate enhancement results from a lower barrier for a conformational and/or quaternary structural change so that they are no longer rate limiting. Consistent with this hypothesis, the PCR analysis suggests that the hydrodynamic changes associated with LLPS-induced oligomerization are more important for stimulation of the H3K4 di-methylation and trimethylation activities but less so for that of the H3K4 monomethylation activity. The fact that these variables could not be deconvoluted to explain LLPS-associated variability in the monomethylation reaction suggests the rate of monomethylation may be limited by mechanisms associated with the di-methylation and trimethylation rates. Such a model is consistent with data indicating that monomethylation occurs in the preformed SET domain (15), whereas multiple methylation depends on a cryptic second active site that is formed only when MLL1 assembles with WRAD_2_ subunits (15, 29, 53, 54). However, this analysis does not rule out the possibility that at least part of the stimulated enzymatic activity upon MLL1 core complex oligomerization may be from the reduced diffusion distance between product release from one active site and the next substrate encounter, which is consistent with reduced apparent *K*_*m*_ values for substrates. Furthermore, it is also possible that the increased activity could be due to an altered arrangement or stoichiometry of the MLL1 core complex. Further experiments will be required to distinguish among these hypotheses. It is of interest to note that in the absence of a crowding agent, LLPS-associated hydrodynamic and enzymatic activity changes begin at physiological ionic strength and increase exponentially as ionic strength is reduced. This suggests a plausible regulatory mechanism in which small changes in ionic strength, possibly through compartmentalization, could have a large impact on MLL1 core complex activity in the cell.

**Figure 9:**
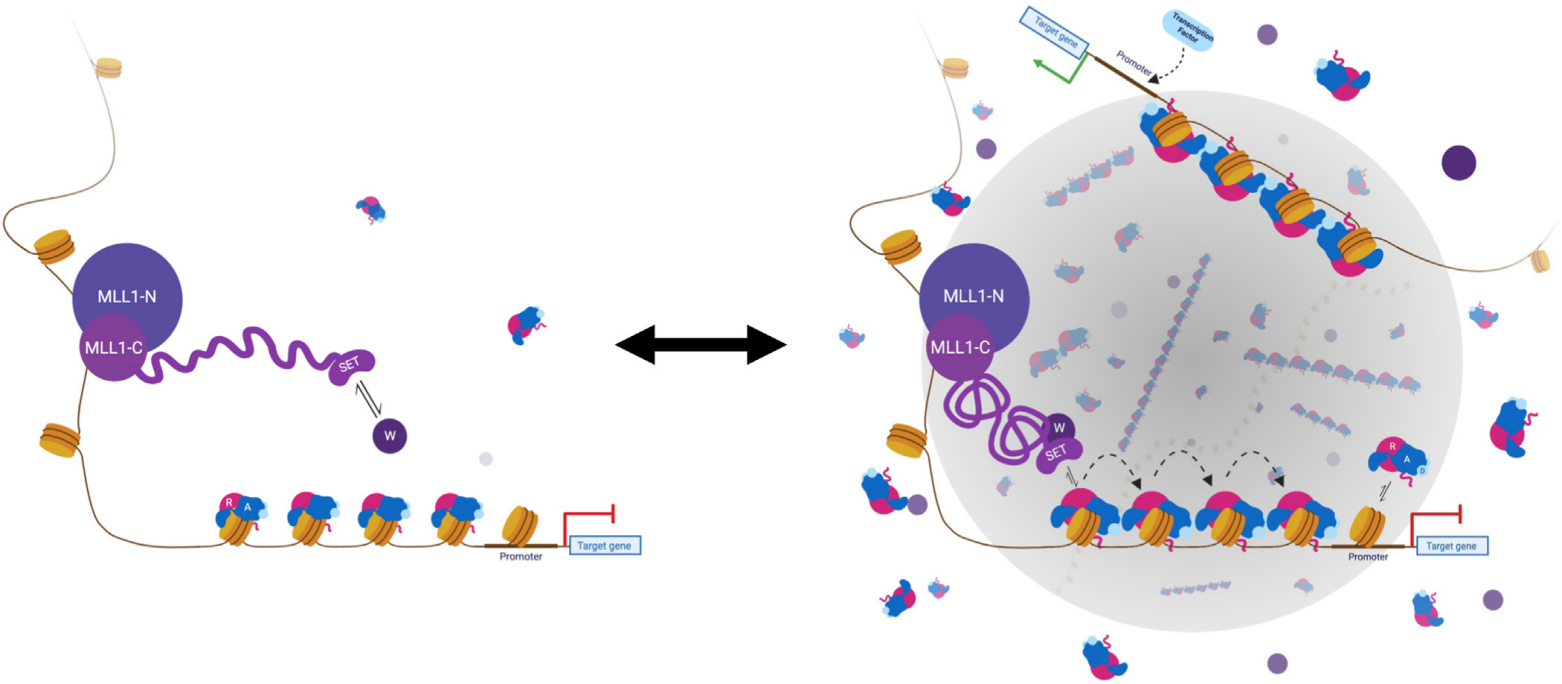
**Phase separation model for spatiotemporal regulation of MLL1 core complex assembly and enzymatic activity within a transcription factory.** (*Left image*) MLL1 N-terminal region (MLL1-N) binds to DNA using its DNA and chromatin-recognition domains. The C-terminal region (MLL1-C), which contains the SET domain, binds to WDR5 (W) to create the MW subcomplex. The RbBP5, Ash2L, DPY-30 (RAD_2_) subcomplex binds nucleosomes. Upon formation of a transcription factory (*right image*), the high local concentration promotes assembly and oligomerization of the MLL1 core complex, stimulating its enzymatic activity. We hypothesize, based on the limited stoichiometry of MLL1 compared to WRAD_2_ components in the cell, that the MW subcomplex functions as a “swinging domain” that successively interacts with adjacent nucleosomes to allow spreading of the H3K4 methylation mark. This methylation results in a more permissive chromatin environment that allows recruitment of transcription factors that, in turn, recruit RNA polymerase II for transcription initiation. Dissolution of the factory imposes a high kinetic barrier for assembly of MWRAD_2_, preventing ectopic methylation. This figure was created with BioRender.com. H3K4, histone H3 lysine 4; MLL, Mixed lineage leukemia; SET, Suppressor of Variegation, Enhancer of Zeste, Trithorax.

Based on these data, we propose a model in which MLL1 enzymatic activity is regulated in the cell at the level of scaffold assembly within a phase-separated transcription factory. Several lines of experimental evidence are consistent with this hypothesis. A common feature of proteins that undergo phase separation include primary sequences with regions of low complexity, or intrinsically disordered regions, that provide the numerous transient multivalent interactions involved in liquid-liquid demixing (27). Examination of the primary sequence of MLL1 by IUPred (55) reveals that the majority of its sequence is predicted to be intrinsically disordered (Fig. 1*A*). In addition, the MLL1 construct used in this investigation and each WRAD_2_ subunit shows significant regions of predicted disorder (Figs. 1*A* and S2; Table S7). Furthermore, use of the PScore (34) and CatGRANULE (35) LLPS prediction programs show that full-length MLL1, as well as all human MLL family proteins, have high phase separation probabilities (Table S4), as does Ash2L and Ash2L-containing subcomplexes (Table S1). However, while the truncated MLL1 construct used in this investigation does not appear to drive LLPS of the MLL1 core complex, it is possible that it functions as a client protein that requires interaction with WDR5 for delivery into a biomolecular condensate (Fig. 9). In addition, the full-length MLL1 protein displays a punctate distribution within mammalian cell nuclei (28), which is another common feature of proteins that undergo LLPS (20). While use of the full-length MLL1 protein for these studies would be ideal, given this previously observed propensity to form puncta, it currently remains infeasible to recombinantly express and purify such a large protein (3969 a.a.) or to isolate it from cell nuclei in sufficient quantities for *in vitro* biophysical analysis. We hypothesize, based on this previous observation, that addition of full-length MLL1 to the core complex would enhance both the LLPS formation and, by extension, the methyltransferase activity of MWRAD_2_, but it remains to be determined the extent to which the low complexity regions not included in the MLL1 construct used in this investigation impact both the punctate pattern in mammalian cells and the activity. Lastly, peptides derived from WDR5 and DPY30, the two most abundant MLL1 core complex subunits (47), were found in proteomic analyses of purified RNA polymerase (Pol) II transcription factories (See Table S1 in reference (56)), which are protein-rich membrane-less compartments involved in spatiotemporal transcriptional control at discrete sites within the genome (57, 58). These results are consistent with studies showing colocalization of MLL1 and RNA Pol II, another protein that undergoes phase separation (59), at the promoters and ORFs of transcriptionally active genes (60, 61).

Combining our results on the assembly of the catalytic module with the observation that it follows a large region of predicted intrinsic disorder in the primary sequence of MLL1 (Fig. 1*A*), we propose a “swinging domain” model for the mechanism of action of the MLL1 core complex within cellular transcription factories (Fig. 9). Swinging domains are a common feature of enzyme complexes involved in multistep assembly pathways and are characterized by a structured mobile domain tethered to other components by conformationally flexible linker regions (62). This may explain why the low complexity region is present not only among MLL1 orthologs but also in the primary sequences in all human SET1 family members, with the main differences being the length of the linker regions that precede the SET domain (Fig. S1 and Table S6). This observation suggests that a swinging domain may be a conserved feature of SET1 family complexes, and linker length differences could be a unique regulatory feature that limits the range of nucleosomes that can be reached within different transcriptional compartments. This hypothesis deserves further investigation.

A swinging domain model where the SET domain–WDR5 complex swings to different nucleosomes provides a satisfying explanation for how the relatively few molecules of MLL1 in the cell could methylate multiple nucleosomes in the promoter and ORFs of genes as they move through the transcription factory (Fig. 9). Given that RAD_2_ subunits are relatively abundant in cells and that the RAD_2_ subcomplex interacts with nucleosomes in the absence of the MW subcomplex (manuscript in preparation), concentration of both subcomplexes within a transcription factory could provide the energy required to overcome the thermodynamic barrier for holocomplex formation and oligomerization at physiological temperatures, resulting in activation of the histone methyltransferase activity of the MLL1 core complex. This model provides an elegant “switch-like” mechanism for spatiotemporal control of H3K4 methylation through the rapid formation or dissolution of biomolecular condensates, which would ultimately regulate the hierarchical MLL1 core complex assembly pathway.

## Experimental procedures

### Protein expression

#### MWRA polycistronic expression

MLL1 SET domain (Uniprot #: Q03164) (a.a. 3745–3969), WDR5 (Uniprot #: P61964) (aa. 2–334 with a 6xHis-tobacco etch virus (TEV) recognition site), RbBP5 (Uniprot #: Q15291) (aa. 1–538), and Ash2L (Uniprot#: Q9UBL3-3) (aa. 1–534) (63) combined in the background of the polycistronic vector pST44 (64) was transformed into Rosetta 2 (DE3) pLysS competent cells and plated on LB agar with 50 μg/ml carbenicillin and 20 μg/ml chloramphenicol. A single colony was used to inoculate a 50 ml LB solution in a 250 ml baffled flask and the antibiotic concentrations were maintained for selection purposes. The culture was grown overnight at 30 °C, then four 2.8 l baffled flasks containing 1 l Terrific Broth II with 50 μg/ml carbenicillin and 20 μg/ml chloramphenicol were inoculated with 12 ml apiece of this overnight culture. The 1 l cultures were grown at 37 °C with 200 RPM shaking for 3 to 4 h, until the optical density at 600 nm was ∼ 1.0. The flasks were then moved to 4 °C for 1 h, then 1 ml of 1 M IPTG was added to each liter of culture (final concentration = 1 mM). The flasks were returned to the incubator, now set to 16 °C with 200 RPM shaking, where they expressed the proteins of interest for 20 to 22 h overnight. Cells were harvested the following day, using 1 l plastic centrifuge bottles in a Beckman centrifuge at 4000*g* for 30 min at 4 °C. The four pellets were resuspended together in 25 ml of new LB media and moved to a 50 ml conical vial. Cells were centrifuged again in a Thermo Scientific refrigerated tabletop centrifuge for 45 min at 4000*g* at 4 °C. The supernatant was poured off and the pellet was flash frozen in liquid nitrogen and stored in a −70 °C freezer until the cells could be lysed.

#### DPY-30 expression

Human DPY-30 (Uniprot #: Q9C005) (a.a. 1–99), in the pHis parallel expression vector (65), was transformed into Rosetta II (DE3) pLysS cells and plated on LB agar with 50 μg/ml carbenicillin and 20 μg/ml chloramphenicol. A single colony was used to inoculate a 50 ml LB Broth in a 250 ml baffled flask that was grown at 30 °C overnight. The next day, 10 ml of the overnight culture was used to inoculate 1 l of TB II media containing 50 μg/ml carbenicillin and 20 μg/ml chloramphenicol in a 2.8 l baffled flask, which was then grown at 37 °C with 200 RPM shaking for 3 to 4 h until an A_600_ of ∼ 1.0. The culture was moved to 4 °C for 1 h, then 1 mM final concentration of IPTG was added and the culture was returned to the 37 °C incubator at 200 RPM shaking to express for an additional 3 h. The cells were then harvested and the pellet was snap frozen with liquid nitrogen and stored in a −70 °C freezer until it could be lysed.

### Protein purification

#### MWRA purification

Prior to lysing, the cell pellet was removed from the −70 °C freezer and thawed in room temperature water for ∼ 1 h. While the pellet was thawing, lysis buffer was prepared (50 mM Tris–HCl, pH 7.5; 300 mM NaCl; 30 mM imidazole; 3 mM DTT, and 1 μM ZnCl_2_, supplemented with one tablet of EDTA-free protease inhibitor cocktail). The pellet was resuspended in at least 50 ml of lysis buffer on ice and then passed through a microfluidizer once to lyse the cells. The lysate was instantly returned to ice for 30 min and then poured into 50 ml round-bottomed centrifuge tubes and cleared in a Beckman floor centrifuge in a JA-20 titanium rotor at 17,000 RPM for 30 min at 4 °C. The cleared supernatant was decanted into a graduated cylinder and then brought up to 250 ml with buffer 1 (same recipe as lysis buffer but without the protease inhibitor cocktail tablet) and loaded onto two tandem HisTrap 5 ml columns on an AKTA purifier, with a flow rate of 0.5 ml/min. The column was washed with 10-column volumes (CV) of buffer 1, followed by elution with a 25 CV linear gradient between 0% and 100% buffer 2 (same recipe as buffer 1 but with 500 mM imidazole). Fractions containing complex components, as determined by SDS-PAGE, were combined, concentrated to 50 ml with a 10,000 molecular weight cut-off (MWCO) Amicon spin concentrator, and then mixed with GST-6xhistidine-TEV protease (final concentration of ∼ 0.1 mg/ml). The mixture was then placed in 3500 MWCO dialysis tubing and dialyzed against 1 l buffer 1 for at least 6 h, with two additional 1 l buffer changes separated by at least 6 h. Dialyzed sample was then loaded over a freshly cleaned tandem HisTrap column and the flowthrough containing TEV-cleaved complex was collected. The flowthrough fractions were pooled and concentrated to ∼15 ml, then passed over a size-exclusion column (Superdex 200 (16/60); 120 ml CV) pre-equilibrated with buffer 3 (20 mM Tris–HCl, pH 7.5; 300 mM NaCl; 1 mM TCEP, and 1 μM ZnCl_2_), with 5 ml sequential injections, eluting into the same fraction tubes. The fractions containing stoichiometric MWRA (assessed by SDS-PAGE) were concentrated to ∼1 mg/ml with the concentration estimated using the extinction coefficient at 280 nm (248,954 M^−1^ cm^−1^) in preparation for DPY-30 addition.

#### DPY-30 purification

6xHIS-DPY-30 was purified following the same protocol described above through lysis and the first two affinity purification steps. DPY-30 was concentrated after the second HisTrap affinity run down ∼2 to 3 mg/ml in a 5 ml volume, followed by size-exclusion chromatography on a Superdex 75 16/60 column in buffer 3. Fractions containing DPY-30 were concentrated to ∼10 mg/ml, aliquoted, and flash-frozen in liquid nitrogen for storage in the −70 °C.

#### MWRAD_2_ assembly and purification

A two-fold molar excess of DPY-30 was mixed with purified MWRA and incubated on ice for 30 min. The new 5-member MWRAD_2_ complex was then passed over a Superdex 200 (16/60) column pre-equilibrated with buffer 3. Fractions containing stoichiometric complex were combined, concentrated with a 30,000 MWCO Amicon concentrator to ∼ 12 mg/ml, aliquoted, flash-frozen in liquid nitrogen, and stored at −70 °C until ready for use.

### Sedimentation velocity-analytical ultracentrifugation

#### Experimental procedures

SV-AUC of the MLL1 core complex was performed using a Beckman-Coulter Proteomelab XL-A analytical ultracentrifuge equipped with absorbance optics. Samples were centrifuged prior to the run at 15,000 RPM for 15 min at 4 °C to remove any aggregates and then loaded into cells containing either a 3 mm or 12 mm epon charcoal centerpiece and sapphire or quartz windows. Cells were placed in an An60 4-hole titanium rotor, previously equilibrated to the run temperature for at least 2 h. Once returned to the chamber, the rotor and cells were equilibrated for an additional 3 to 4 h. Once equilibrated, a sedimentation velocity run at 50,000 RPM for 200 scans was selected, with the time interval between scans set to zero. An initial wavelength scan at 3000 RPM was performed to confirm that the wavelength of interest was between 0.1 to 1 AU for each sample; then the ramp-up to 50,000 RPM was begun, without stopping in between. The first few scans of each cell were observed to check for leaks in the cells and then the run was allowed to proceed overnight.

#### Data analysis

SV-AUC results were analyzed in the software SEDFIT (https://sedfitsedphat.github.io) (66), as previously described (17). Briefly, raw radial absorbance scans (∗.ra) were loaded into SEDFIT, leaving out any extra scans near the end of the run, when the complex was fully sedimented and absorbance signal across the entire sector was ∼0. The “continuous c(s) distribution” model was selected for analysis and the meniscus and bottom of the cell was positioned by eye. The range of desired s-values was selected (normally 10), as well as the resolution (normally 10 points/s-value). An initial analysis was performed to optimize linear parameters, and then the meniscus and frictional ratio were allowed to float and fit using Simplex and Marquardt-Levenberg nonlinear least squares regression algorithms. RMSD values were between 0.003 and 0.01 for all data. Density, viscosity, and partial specific volume values were estimated by inputting buffer components and amino acid sequences into the program SEDNTERP (67). The values obtained are listed in Table S6 for reference (17). *c(s)* distributions were displayed and integrated using GUSSI (68).

For the *c(s,f*_*r*_*)* analysis, the data were first imported into SEDFIT with reduced radial resolution (0.006 cm compared to the default 0.003 cm) and loading every second scan, to reduce the computational power required (69). These were fit using the *c(s, f*_*r*_*)* method in SEDFIT with resolutions of 50 for both the sedimentation coefficient and frictional ratio dimensions.

### Methyltransferase activity assay

The H3K4 methyltransferase activity of the MLL1 core complex was assayed using a label-free quantitative MALDI-TOF mass spectrometry assay (15), using H3 peptide (residues 1–20, with an additional C-terminal GGK-biotin moiety) as substrate. The reaction buffer was 50 mM Tris, pH 9.0; 5% (v/v) glycerol; 1 μM ZnCl_2_; 3 mM DTT; and variable concentrations of NaCl ranging from 25 to 300 mM. Five micromolar of MWRAD_2_ was combined with 10 μM H3 peptide and 250 μM SAM in this buffer and allowed to react at the desired reaction temperature for 24 h. At various timepoints, 2 μl of the reaction volume were removed and quenched in 2 μl of 1% TFA and stored at −20 °C until they could be analyzed. The quenched timepoints were mixed 1:4 with α-cyano-hydroxycinnamic acid and then 2 μl of this mixture was spotted on a ground steel 384-spot target plate and allowed to dry for at least 2 h. A Bruker Autoflex III MALDI-TOF mass spectrometer in reflectron mode was used to obtain spectra for each time point, which was the average of 1000 laser shots per spot. Peaks corresponding to unmethylated and each methylation state of the peptide were integrated and summed to determine the total intensity. The relative intensity of each methylation state was then determined by dividing the intensity of each methylation state by the total intensity. The concentration of each methylation state at each time point was determined by multiplying the relative intensity of each species by the total peptide concentration. At least two technical replicates were assayed for each [NaCl].

### Single turnover condensate activity assay

#### Experimental procedures

The MALDI reaction buffer listed above, with either 200 mM or 50 mM NaCl, was placed in a 5 mm:3 mm reducer tube with a final concentration of 675 μM ^13^C-SAM (Sigma-Aldrich) (in 10 mM HCl) and 100 μM H3 peptide (in distilled H_2_O). Initial tuning, matching, shimming, and pulse calibration were performed on this sample in a Bruker Avance NEO 600 MHz spectrometer, similar to (39). After an initial ^1^H-^13^C-HSQC, MWRAD_2_ was added to a final concentration of 5 μM to initiate the reaction. The final volume of the reaction was 200 μl. The reaction was allowed to proceed for 48 scans (∼90 min at 2 min/scan) at 298 K (25 °C), and then the sample was removed from the magnet and the NMR tube, placed in a 1.7 ml microcentrifuge tube, and spun down at 16,000*g* at 25 °C for 10 min. The supernatant from this centrifugation was collected (190 μl) and returned to the same 5:3 reducer tube (which was cleaned during the centrifugation step) and returned to the NMR. After an initial HSQC, 10 μl each of stock H3 peptide (2 mM) and ^13^C-SAM (4.5 mM) was added to provide sufficient substrate for continued turnover and the reaction was again monitored for another 48 scans. Based on the simulations in KinTek Explorer software (40) (see below), concentrations of ^13^C-SAM were chosen to minimize product inhibition by the cofactor product SAH.

### Fitting in KinTek explorer

KinTek explorer version 11.0.1.3 (40) was used for simulation and global fitting single turnover condensate activity reaction data using model 4B. The simulation was modeled exactly as performed by inputting experimental details and reaction concentrations, including incorporation of a mixing step after the first 90-min reaction with the appropriate dilution factor to account for the addition of H3 peptide and ^13^C-SAM for the dilute phase assay. Experimental intensities were modeled using scaling factors as follows:

a∗(me1)+bkg1

b∗(2∗me2)+bkg2

c∗(3∗me3)+bkg3

Where *a*, *b*, and *c* scales ^13^C-signal relative to each respective H3 peptide concentration and relative to background for each species (*bkg1-3*). Turnover numbers (*k*_*cat*_*)* for the mono- (*k*_*me1*_) and di-methylation (*k*_*me2*_) reactions were fit for the bulk phase experiments and the resulting values were used for both bulk and dilute phases and locked during global fitting. The *k*_*cat*_*/K*_*m*_ parameter was derived by assuming that all bound peptide substrate is converted to product for each reaction by setting the dissociation rate to zero and then fitting for the corresponding association rate while all other rate constants were fixed at nonrate limiting values. The enzymatic partition coefficient (*K*_*p*_^*enz*^) is defined as the ratio of rate constants that define the equilibrium between the phase-separated state and the dilute state of the enzyme as shown in Figure 4*B*. The value of *K*_*p*_^*enz*^ was determined by fixing the reverse microscopic rate constant to a nonrate limiting value (1000 min^−1^) and fitting for the forward microscopic rate constant.

### Labeling and assembly of fluorescent MWRAD_2_ complexes

Recombinant WDR5 or RbBP5 were expressed and purified as previously described (29). Purified proteins at ∼14 mg/ml were dialyzed into labeling buffer composed of 20 mM Hepes, pH 7.0, 300 mM NaCl, 1 mM TCEP, and 1 μM ZnCl_2_. The neutral pH was chosen to facilitate selective labeling of the free amino terminus of the protein, which has a lower *pK*_*a*_ than the primary amines of the lysine side chains (70). The protein was mixed with AlexaFluor 488 NHS Ester (Invitrogen) in a 1:6 (for WDR5) or 1:5 (for RbBP5) molar excess of label and reacted for 3 h at 4 °C. The entire reaction volume for each protein was then loaded onto a Superdex 200 10/300 Gl size-exclusion column (GE) to separate the labeled protein from the unreacted fluorophore. The labeled protein fractions were then combined and concentrated by ultrafiltration in a 10,000 MWCO concentrator (Millipore). Once concentrated, the degree of labeling was determined using the equations shown below:

$$A_{protein}=A_{280}-A_{\max}*\left(correction\;factor\right)$$

$$A_{protein}∕\left(pathlength*\varepsilon_{protein}\right)=\left[protein\right]$$

$$Degree\;of\;labeling=\left(A_{\max}*\left(protein\;MW\right)\right)∕\left(\left[protein\right]*\varepsilon_{dye}\right)$$

The degree of labeling for WDR5 (W∗) was found to be 1.1 or ∼1 molecule of fluorophore for each molecule of WDR5. The degree of labeling determined for RbBP5 (R∗) was 1.9 or ∼2 molecules of fluorophore per molecule of RbBP5. Each labeled protein was then mixed in equivalent molar ratios with the other recombinant, unlabeled complex components and loaded onto a Superdex 200 10/300 Gl size-exclusion column, and fractions containing stoichiometric complex were pooled, concentrated, and stored at −70 °C until use.

### LLPS assays

MWRAD_2_ at a concentration of 5 μM was mixed with H3^1–20^ peptide (100–500 μM) and 250 μM SAM in either physiological (∼100–150 mM) or subphysiological (∼25–50 mM) NaCl buffers containing 50 mM Tris, pH 9.0, 1 μM ZnCl_2_, 3 mM DTT, and 5% (w/v) glycerol in the presence or absence of 3 to 7% (w/v) dextran sulfate (avg. M.W. = 500,000 Da) as a crowding agent. One microliter of each sample was pipetted into the depression of a 12-well precleaned frosted end Bioworld microscope slide, covered by a cover slip, and observed on a Zeiss light microscope in DIC mode at 40× magnification. Single images and movies were taken using a Hamamatsu camera connected to the microscope. All images taken are of samples at room temperature (∼23 °C). In addition to DIC, M(W∗)RAD_2_ or MW(R∗)AD_2_ were imaged with the FITC filter activated. As a control for phase separation, reaction mixtures were compared in the presence and absence of 5% 1,6-hexanediol.

### Statistical methods

Pearson’s linear correlation coefficient was used to assess relationships between H3K4 methylation rates and the biophysical parameters. The significance of the Pearson correlation coefficient was evaluated using a *t* test in XLStat software (https://www.xlstat.com/en/).

PCR provides a useful method to determine impact of each parameter on H3K4 methylation rates. This involved first using principal component analysis on the independent variables, ionic strength, *S*_*w*_, LLPS score, % monomer, and % oligomer. Linear combinations of the covarying parameters were grouped into new variables, called principal components, that were then used in regression analyses to explain variability of H3K4 methylation rates. Principle components chosen for the regression analyses were determined using ANOVA to select the best regression models that explained at least 80% of the data variability. Transformation of the resulting regression vectors back to the scale of the input parameters revealed the relative contributions of each parameter to the variability in H3K4 methylation rates. The *S*_*w*_ value was determined by integrating *c(s)* plots in SV-AUC experiments between 0 and 30 S to determine the signal weight-average sedimentation coefficient. LLPS score was determined by manually counting the number of LLPS droplets that could be observed in DIC microscopy images (Fig. 6). % monomer is the relative amount of holo-MLL1 core complex sedimenting between S-values 6.8 and 7.6 in c(s) SV-AUC plots. % oligomer is the relative amount of signal observed in SV-AUC plots sedimenting between 7.6-30 *S*. The raw data from these experiments are summarized in Table S5. All parameters were standardized to have a mean of zero and an SD of 1 to place them on the same scale using the z-score method of subtracting the mean and dividing by the SD of each value of each parameter. Eigenvectors ≥(±) 0.5 after rounding were considered significant. Calculations were performed using XLStat software and plotted using Prism version 9.

## Data availability

SV-AUC, MALDI-TOF mass spectrometry spectra, NMR spectra, and microscopy images are available upon reasonable request.

## Supporting information

This article contains supporting information (34, 35, 55).

## Conflicts of interest

M. S. C. owns stock and serves on the Consultant Advisory Board for Kathera Bioscience Inc, the makers of antifungal technologies. M. S. C. also hold US patents (8133690), (8715678), and (10392423) for compounds and methods for inhibiting SET1/MLL family complexes. The other authors declare no competing financial interests.

## References

[1] Liu C.L., Kaplan T., Kim M., Buratowski S., Schreiber S.L., Friedman N. (2005). Single-nucleosome mapping of histone modifications in S. Cerevisiae. PLoS Biol..

[2] Pokholok D.K., Harbison C.T., Levine S., Cole M., Hannett N.M., Lee T.I. (2005). Genome-wide map of nucleosome acetylation and methylation in yeast. Cell.

[3] Santos-Rosa H., Schneider R., Bannister A.J., Sherriff J., Bernstein B.E., Emre N.C. (2002). Active genes are tri-methylated at K4 of histone H3. Nature.

[4] Wysocka J., Swigut T., Xiao H., Milne T.A., Kwon S.Y., Landry J. (2006). A PHD finger of NURF couples histone H3 lysine 4 trimethylation with chromatin remodelling. Nature.

[5] Santos-Rosa H., Schneider R., Bernstein B.E., Karabetsou N., Morillon A., Weise C. (2003). Methylation of histone H3 K4 mediates association of the Isw1p ATPase with chromatin. Mol. Cell.

[6] Sims R.J., Chen C.F., Santos-Rosa H., Kouzarides T., Patel S.S., Reinberg D. (2005). Human but not yeast CHD1 binds directly and selectively to histone H3 methylated at lysine 4 via its tandem chromodomains. J. Biol. Chem..

[7] Pray-Grant M.G., Daniel J.A., Schieltz D., Yates J.R., Grant P.A. (2005). Chd1 chromodomain links histone H3 methylation with SAGA- and SLIK-dependent acetylation. Nature.

[8] Kim T., Buratowski S. (2009). Dimethylation of H3K4 by Set1 recruits the Set3 histone deacetylase complex to 5' transcribed regions. Cell.

[9] Cheng J., Blum R., Bowman C., Hu D., Shilatifard A., Shen S. (2014). A role for H3K4 monomethylation in gene repression and partitioning of chromatin readers. Mol. Cell.

[10] van Dijk K., Marley K.E., Jeong B.R., Xu J., Hesson J., Cerny R.L. (2005). Monomethyl histone H3 lysine 4 as an epigenetic mark for silenced euchromatin in Chlamydomonas. Plant Cell.

[11] Bryk M., Briggs S.D., Strahl B.D., Curcio M.J., Allis C.D., Winston F. (2002). Evidence that Set1, a factor required for methylation of histone H3, regulates rDNA silencing in S. cerevisiae by a Sir2-independent mechanism. Curr. Biol..

[12] Krogan N.J., Dover J., Khorrami S., Greenblatt J.F., Schneider J., Johnston M. (2002). COMPASS, a histone H3 (Lysine 4) methyltransferase required for telomeric silencing of gene expression. J. Biol. Chem..

[13] Nislow C., Ray E., Pillus L. (1997). SET1, a yeast member of the trithorax family, functions in transcriptional silencing and diverse cellular processes. Mol. Biol. Cell.

[14] Shilatifard A. (2012). The COMPASS family of histone H3K4 Methylases: mechanisms of regulation in development and disease pathogenesis. Annu. Rev. Biochem..

[15] Patel A., Dharmarajan V., Vought V.E., Cosgrove M.S. (2009). On the mechanism of multiple lysine methylation by the human mixed lineage leukemia protein-1 (MLL1) core complex. J. Biol. Chem..

[16] Dou Y., Milne T.A., Ruthenburg A.J., Lee S., Lee J.W., Verdine G.L. (2006). Regulation of MLL1 H3K4 methyltransferase activity by its core components. Nat. Struct. Mol. Biol..

[17] Namitz K.E.W., Tan S., Cosgrove M.S. (2023). Hierarchical assembly of the MLL1 core complex regulates H3K4 methylation and is dependent on temperature and component concentration. J. Biol. Chem..

[18] Rieder D., Trajanoski Z., McNally J.G. (2012). Transcription Factories. Front. Genet..

[19] Li P., Banjade S., Cheng H.C., Kim S., Chen B., Guo L. (2012). Phase transitions in the assembly of multivalent signalling proteins. Nature.

[20] Banani S.F., Lee H.O., Hyman A.A., Rosen M.K. (2017). Biomolecular condensates: organizers of cellular biochemistry. Nat. Rev. Mol. Cell Biol..

[21] Strulson C.A., Molden R.C., Keating C.D., Bevilacqua P.C. (2012). RNA catalysis through compartmentalization. Nat. Chem..

[22] Koga S., Williams D.S., Perriman A.W., Mann S. (2011). Peptide-nucleotide microdroplets as a step towards a membrane-free protocell model. Nat. Chem..

[23] Dhar A., Samiotakis A., Ebbinghaus S., Nienhaus L., Homouz D., Gruebele M. (2010). Structure, function, and folding of phosphoglycerate kinase are strongly perturbed by macromolecular crowding. Proc. Natl. Acad. Sci. U. S. A..

[24] Pozdnyakova I., Wittung-Stafshede P. (2010). Non-linear effects of macromolecular crowding on enzymatic activity of multi-copper oxidase. Biochim. Biophys. Acta.

[25] Aumiller W.M., Davis B.W., Hatzakis E., Keating C.D. (2014). Interactions of macromolecular crowding agents and cosolutes with small-molecule substrates: effect on horseradish peroxidase activity with two different substrates. J. Phys. Chem. B.

[26] Pastor I., Vilaseca E., Madurga S., Garces J.L., Cascante M., Mas F. (2011). Effect of crowding by dextrans on the hydrolysis of N-Succinyl-L-phenyl-Ala-p-nitroanilide catalyzed by alpha-chymotrypsin. J. Phys. Chem. B.

[27] Mitrea D.M., Kriwacki R.W. (2016). Phase separation in biology; functional organization of a higher order. Cell Commun. Signal..

[28] Yano T., Nakamura T., Blechman J., Sorio C., Dang C.V., Geiger B. (1997). Nuclear punctate distribution of ALL-1 is conferred by distinct elements at the N terminus of the protein. Proc. Natl. Acad. Sci. U. S. A..

[29] Patel A., Vought V.E., Dharmarajan V., Cosgrove M.S. (2008). A conserved arginine-containing motif crucial for the assembly and enzymatic activity of the mixed lineage leukemia protein-1 core complex. J. Biol. Chem..

[30] Alberti S., Saha S., Woodruff J.B., Franzmann T.M., Wang J., Hyman A.A. (2018). A user's guide for phase separation assays with purified proteins. J. Mol. Biol..

[31] Vedadi M., Blazer L., Eram M.S., Barsyte-Lovejoy D., Arrowsmith C.H., Hajian T. (2017). Targeting human SET1/MLL family of proteins. Protein Sci..

[32] Schuck P. (1998). Sedimentation analysis of noninteracting and self-associating solutes using numerical solutions to the Lamm equation. Biophys. J..

[33] Kroschwald S., Maharana S., Simon A. (2017). Hexanediol: a chemical probe to investigate the material properties of membrane-less compartments. Matters.

[34] Vernon R.M., Chong P.A., Tsang B., Kim T.H., Bah A., Farber P. (2018). Pi-Pi contacts are an overlooked protein feature relevant to phase separation. Elife.

[35] Bolognesi B., Lorenzo Gotor N., Dhar R., Cirillo D., Baldrighi M., Tartaglia G.G. (2016). A concentration-dependent liquid phase separation can cause toxicity upon increased protein expression. Cell Rep..

[36] Rahman S., Hoffmann N.A., Worden E.J., Smith M.L., Namitz K.E.W., Knutson B.A. (2022). Multistate structures of the MLL1-WRAD complex bound to H2B-ubiquitinated nucleosome. Proc. Natl. Acad. Sci. U. S. A..

[37] Xue H., Yao T., Cao M., Zhu G., Li Y., Yuan G. (2019). Structural basis of nucleosome recognition and modification by MLL methyltransferases. Nature.

[38] Park S.H., Ayoub A., Lee Y.T., Xu J., Kim H., Zhang W. (2019). Cryo-EM structure of the human mixed lineage leukemia-1 complex bound to the nucleosome. bioRxiv.

[39] Usher E.T., Namitz K.E.W., Cosgrove M.S., Showalter S.A. (2021). Probing multiple enzymatic methylation events in real time with NMR spectroscopy. Biophys. J..

[40] Johnson K.A., Simpson Z.B., Blom T. (2009). Global kinetic explorer: a new computer program for dynamic simulation and fitting of kinetic data. Anal. Biochem..

[41] Tibble R.W., Depaix A., Kowalska J., Jemielity J., Gross J.D. (2021). Biomolecular condensates amplify mRNA decapping by biasing enzyme conformation. Nat. Chem. Biol..

[42] Peeples W., Rosen M.K. (2021). Mechanistic dissection of increased enzymatic rate in a phase-separated compartment. Nat. Chem. Biol..

[43] Dao T.P., Martyniak B., Canning A.J., Lei Y., Colicino E.G., Cosgrove M.S. (2019). ALS-linked mutations affect UBQLN2 oligomerization and phase separation in a position- and amino acid-dependent manner. Structure.

[44] Larson A.G., Elnatan D., Keenen M.M., Trnka M.J., Johnston J.B., Burlingame A.L. (2017). Liquid droplet formation by HP1alpha suggests a role for phase separation in heterochromatin. Nature.

[45] Brown P.H., Schuck P. (2006). Macromolecular size-and-shape distributions by sedimentation velocity analytical ultracentrifugation. Biophys. J..

[46] Chaton C.T., Herr A.B. (2015). Elucidating complicated assembling systems in biology using size-and-shape analysis of sedimentation velocity data. Methods Enzymol..

[47] van Nuland R., Smits A.H., Pallaki P., Jansen P.W., Vermeulen M., Timmers H.T. (2013). Quantitative dissection and stoichiometry determination of the human SET1/MLL histone methyltransferase complexes. Mol. Cell. Biol..

[48] Zhang P., Chaturvedi C.P., Tremblay V., Cramet M., Brunzelle J.S., Skiniotis G. (2015). A phosphorylation switch on RbBP5 regulates histone H3 Lys4 methylation. Genes Dev..

[49] Wang K.C., Yang Y.W., Liu B., Sanyal A., Corces-Zimmerman R., Chen Y. (2011). A long noncoding RNA maintains active chromatin to coordinate homeotic gene expression. Nature.

[50] Tariq M., Nussbaumer U., Chen Y., Beisel C., Paro R. (2009). Trithorax requires Hsp90 for maintenance of active chromatin at sites of gene expression. Proc. Natl. Acad. Sci. U. S. A..

[51] Mitrea D.M., Cika J.A., Stanley C.B., Nourse A., Onuchic P.L., Banerjee P.R. (2018). Self-interaction of NPM1 modulates multiple mechanisms of liquid-liquid phase separation. Nat. Commun..

[52] Martin E.W., Harmon T.S., Hopkins J.B., Chakravarthy S., Incicco J.J., Schuck P. (2021). A multi-step nucleation process determines the kinetics of prion-like domain phase separation. Nat. Commun..

[53] Patel A., Vought V.E., Dharmarajan V., Cosgrove M.S. (2011). A novel non-SET domain multi-subunit methyltransferase required for sequential nucleosomal histone H3 methylation by the mixed lineage leukemia protein-1 (MLL1) core complex. J. Biol. Chem..

[54] Patel A., Vought V.E., Swatkoski S., Viggiano S., Howard B., Dharmarajan V. (2014). Automethylation activities within the mixed lineage leukemia-1 (MLL1) core complex reveal evidence supporting a "two-active site" model for multiple histone H3 lysine 4 methylation. J. Biol. Chem..

[55] Dosztanyi Z., Csizmok V., Tompa P., Simon I. (2005). The pairwise energy content estimated from amino acid composition discriminates between folded and intrinsically unstructured proteins. J. Mol. Biol..

[56] Melnik S., Deng B., Papantonis A., Baboo S., Carr I.M., Cook P.R. (2011). The proteomes of transcription factories containing RNA polymerases I, II or III. Nat. Methods.

[57] Jackson D.A., Hassan A.B., Errington R.J., Cook P.R. (1993). Visualization of focal sites of transcription within human nuclei. EMBO J..

[58] Wansink D.G., Schul W., van der Kraan I., van Steensel B., van Driel R., de Jong L. (1993). Fluorescent labeling of nascent RNA reveals transcription by RNA polymerase II in domains scattered throughout the nucleus. J. Cell Biol..

[59] Boehning M., Dugast-Darzacq C., Rankovic M., Hansen A.S., Yu T., Marie-Nelly H. (2018). RNA polymerase II clustering through carboxy-terminal domain phase separation. Nat. Struct. Mol. Biol..

[60] Guenther M.G., Jenner R.G., Chevalier B., Nakamura T., Croce C.M., Canaani E. (2005). Global and Hox-specific roles for the MLL1 methyltransferase. Proc. Natl. Acad. Sci. U. S. A..

[61] Milne T.A., Dou Y., Martin M.E., Brock H.W., Roeder R.G., Hess J.L. (2005). MLL associates specifically with a subset of transcriptionally active target genes. Proc. Natl. Acad. Sci. U. S. A..

[62] Perham R.N. (2000). Swinging arms and swinging domains in multifunctional enzymes: catalytic machines for multistep reactions. Annu. Rev. Biochem..

[63] UniProt C. (2019). UniProt: a worldwide hub of protein knowledge. Nucleic Acids Res..

[64] Tan S., Kern R.C., Selleck W. (2005). The pST44 polycistronic expression system for producing protein complexes in Escherichia coli. Protein Expr. Purif..

[65] Sheffield P., Garrard S., Derewenda Z. (1999). Overcoming expression and purification problems of RhoGDI using a family of “parallel” expression vectors. Protein Expr. Purif..

[66] Schuck P. (2000). Size-distribution analysis of macromolecules by sedimentation velocity ultracentrifugation and lamm equation modeling. Biophys. J..

[67] Laue T.M., Shah B., Ridgeway T.M., Pelletier S.L., Harding S.E., Rowe A.J., Horton L.C. (1992). Analytical Ultracentrifugation in Biochemistry and Polymer Science.

[68] Brautigam C.A. (2015). Calculations and publication-quality illustrations for analytical ultracentrifugation data. Methods Enzymol..

[69] Brown P.H., Balbo A., Schuck P. (2007). Using prior knowledge in the determination of macromolecular size-distributions by analytical ultracentrifugation. Biomacromolecules.

[70] Toseland C.P. (2013). Fluorescent labeling and modification of proteins. J. Chem. Biol..

