# Supplementary material for: Phase separation promotes a highly active oligomeric scaffold of the MLL1 core complex for regulation of histone H3K4 methylation
Source: J Biol Chem. 2023 Sep 1;299(10):105204. doi: 10.1016/j.jbc.2023.105204 (PMC10551905; doi:10.1016/j.jbc.2023.105204)
Supplement: Supporting information [file mmc1.pdf]

Supporting Information

Phase Separation Promotes a Highly Active Oligomeric Scaffold of the MLL1 Core Complex for Regulation of H3K4 Methylation

Kevin E. W. Namitz[1, 2], Scott A. Showalter[3], and Michael S. Cosgrove[1]*

[1]State University of New York (SUNY) Upstate Medical University, Department of Biochemistry and Molecular Biology, Syracuse, New York.
[2] Current Address: Penn State University, Department of Chemistry, University Park, Pennsylvania
[3] Penn State University, Department of Chemistry, University Park, Pennsylvania

*To whom correspondence should be addressed: Michael S. Cosgrove, Ph.D., Department of Biochemistry and Molecular Biology, SUNY Upstate Medical University, 750 East Adams Street, Syracuse, NY 13210, Phone: (315) 464-7751, E-mail: cosgrovm@upstate.edu

Figure S1 – Namitz, Showalter and Cosgrove

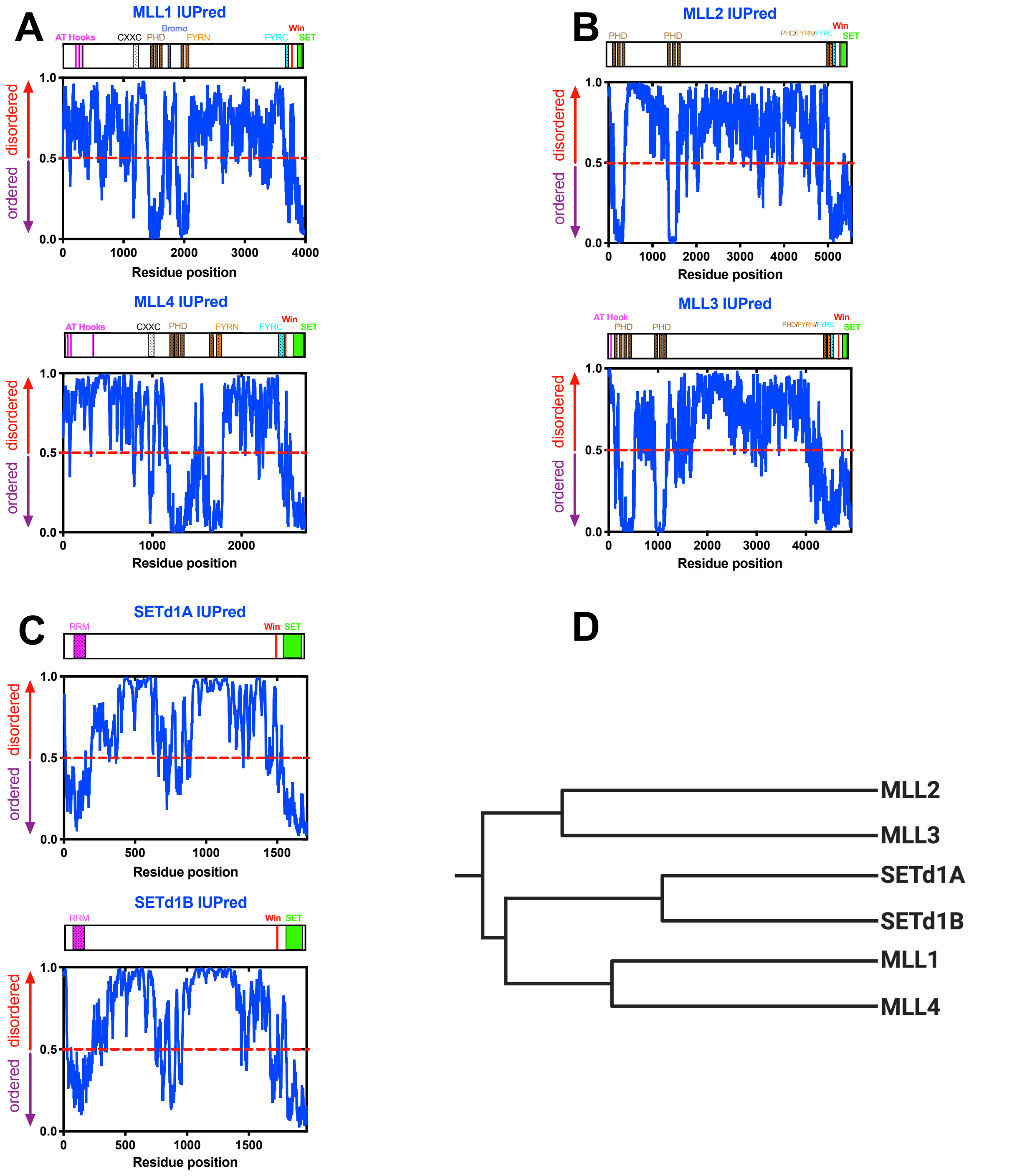

Figure S1: Each human SET1 family member has large predicted intrinsically disordered regions. IUPred disorder prediction (55) for (A), full-length Human MLL1 (Uniprot # Q03164) (top) (note: this panel is reproduced from Figure 1A in the main body of the manuscript) and MLL4 (Uniprot # Q9UMN6) (bottom); (B), MLL2 (Uniprot # O14686) (top) and MLL3 (Uniprot # Q8NEZ4) (bottom); (C), SETd1A (Uniprot # O15047) (top) and SETd1B (Uniprot # Q9UPS6) (bottom). Uniprot sub-domain boundaries are shown in the schematic above each panel and are summarized in Table S6. Note the similarities between the structured domains and linker lengths among the family members from the different phylogenetic clades (MLL1 and MLL4), (MLL2 and MLL3) and (SETd1A and SETd1B). (D) Cladogram showing evolutionary relationships among SET1 family members (created with BioRender.com).

Figure S2 – Namitz, Showalter and Cosgrove

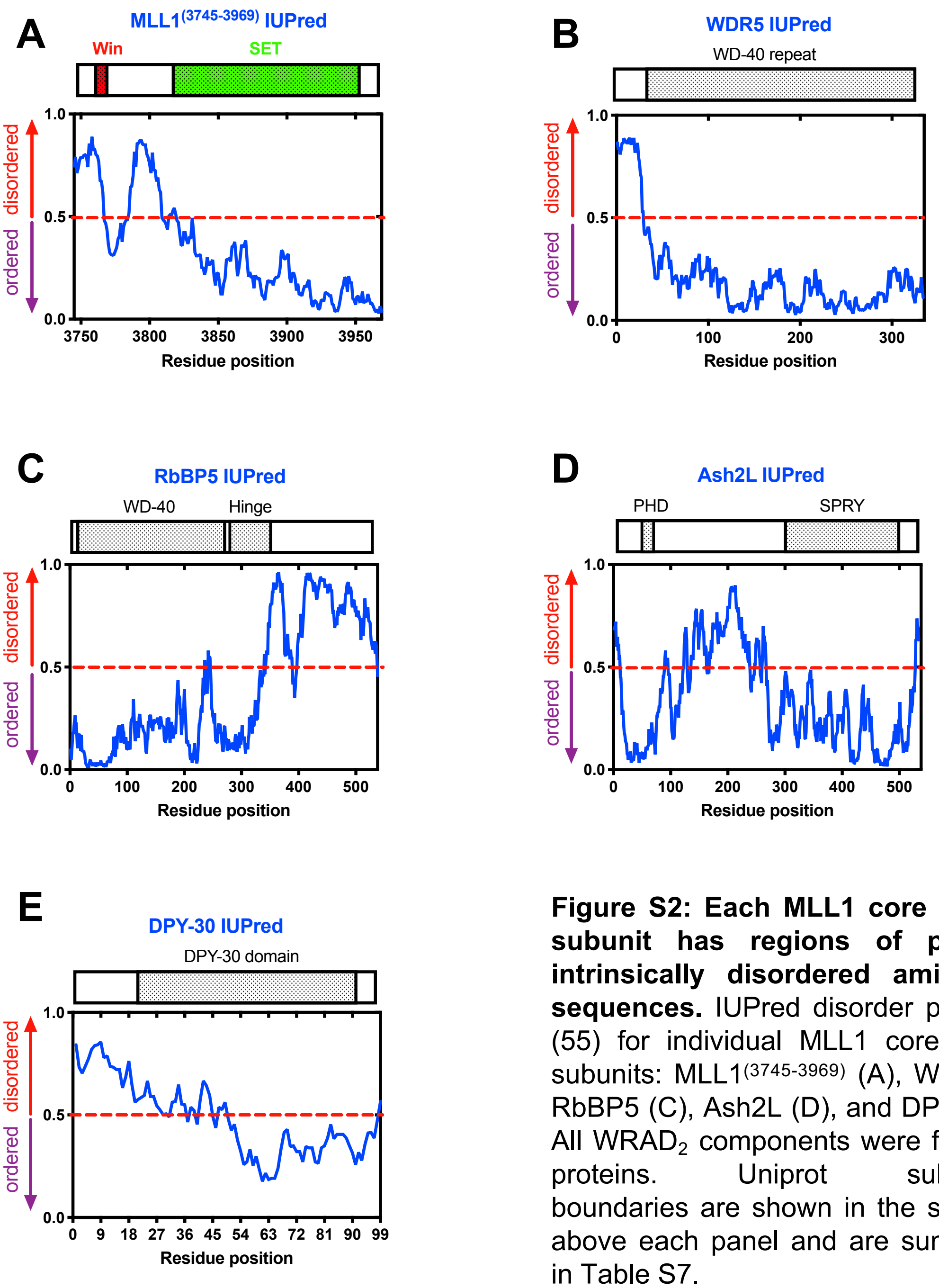

Figure S2: Each MLL1 core complex subunit has regions of predicted intrinsically disordered amino acid sequences. IUPred disorder predictions (55) for individual MLL1 core complex subunits: $MLL1^{(3745\text{-}3969)}$ (A), WDR5 (B), RbBP5 (C), Ash2L (D), and DPY-30 (E). All $WRAD_2$ components were full-length proteins. Uniprot sub-domain boundaries are shown in the schematic above each panel and are summarized in Table S7.

Figure S3 – Namitz, Showalter and Cosgrove

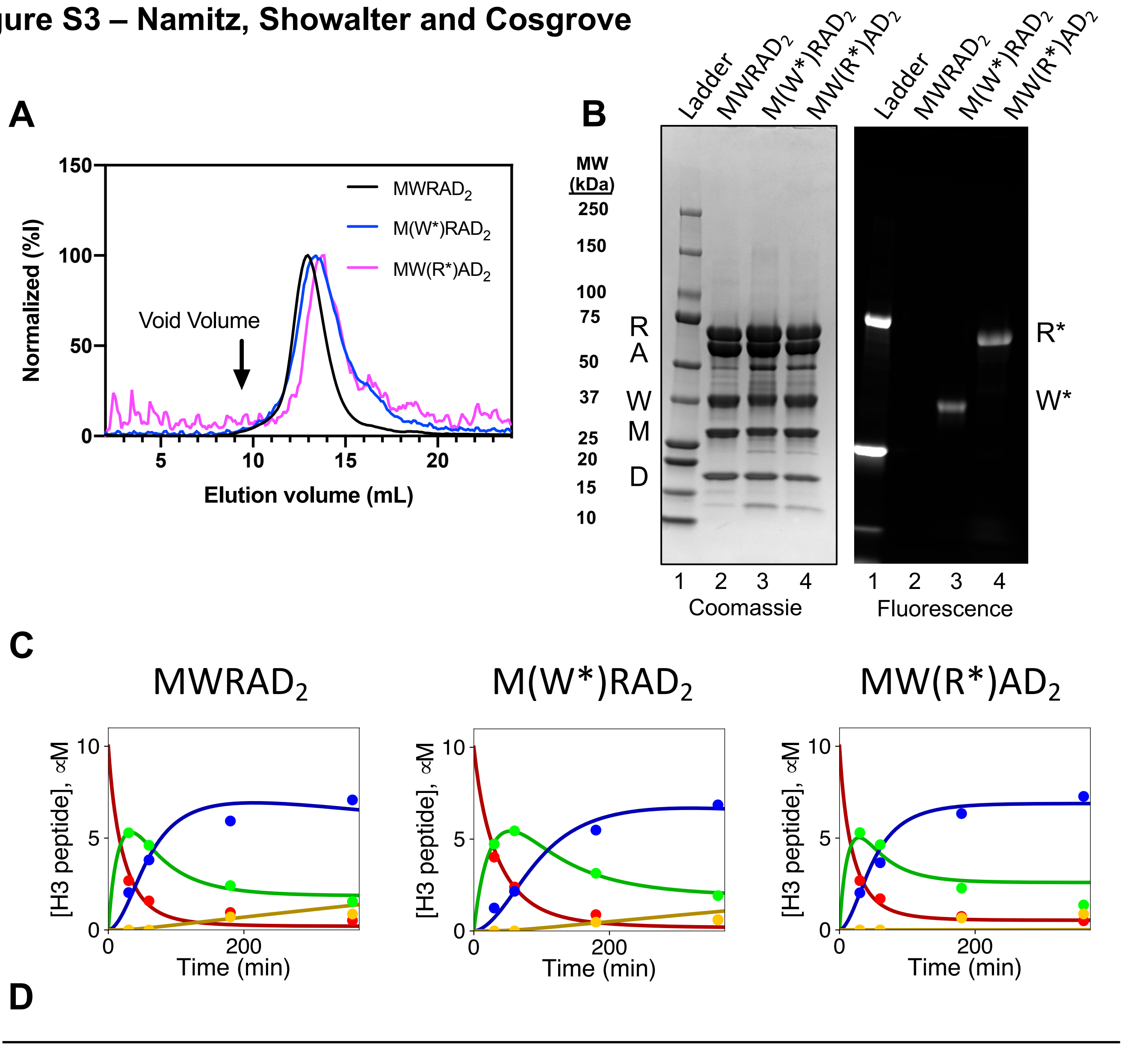

D

Complex	k_{me1}	k_{me2}	k_{me3}	k_{inact}
$MWRAD_2$	0.16 (0.02)	0.12 (0.02)	0.001 (0.002)	0.11 (0.03)
$M(W^*)RAD_2$	0.1 (0.01)	0.07 (0.01)	0.0009 (0.0008)	0.07 (0.01)
$MW(R^*)AD_2$	0.19 (0.02)	0.16 (0.04)	0.000005 (ND)	0.17 (0.04)

Figure S3: Assembly of fluorescently-labeled MLL1 core complexes. Representative subunits from the MW or RAD_2 subcomplexes were chosen to be N-terminally-labeled with AlexaFluor™ 488. WDR5 (W*) or RbBP5 (R*) were labeled as described in Methods and assembled with the other unlabeled complex subunits and purified by SEC (A). The elution profiles show that the holo-complexes assembled with W* (MW^*RAD_2) or R* (MWR^*AD_2) have similar elution profiles to that of the unlabeled complex ($MWRAD_2$). (B) SDS-PAGE of purified complexes visualized by Coomassie blue staining (left panel) or fluorescence imaging (right panel). (C) Reaction progress curves from MALDI-TOF methyltransferase assays comparing the enzymatic activity of unlabeled and labeled complexes at a concentration of 5 μM and 25°C. (D) Summary of pseudo-first order rate constants (+/- S.E.) for unlabeled and labeled complexes.

Figure S4 – Namitz, Showalter and Cosgrove

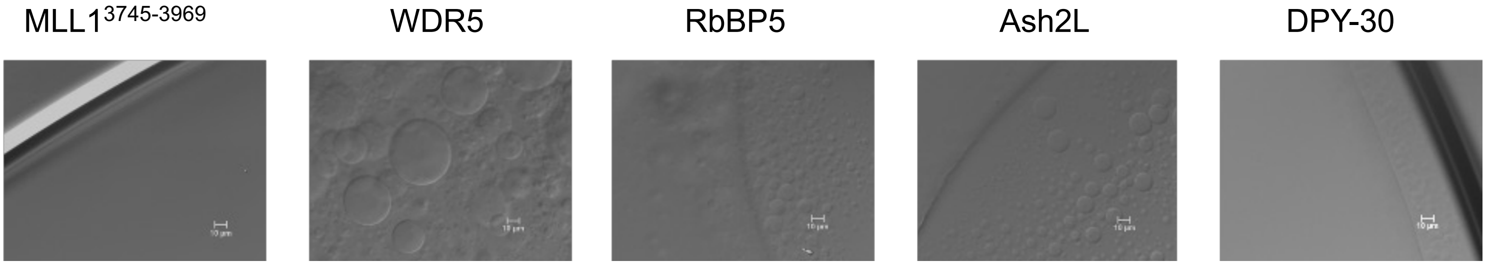

Figure S4: Individual MWRAD$_2$ subunits show varying degrees of LLPS propensity
Individual subunits as labeled above at 5μM, with 5μM H3 peptide, 125μM SAM, 150mM NaCl and 3.5% dextran. LLPS droplets are observed for WDR5. RbBP5 and Ash2l. The scale bar in each image is 10 μm.

Figure S5 – Namitz, Showalter and Cosgrove

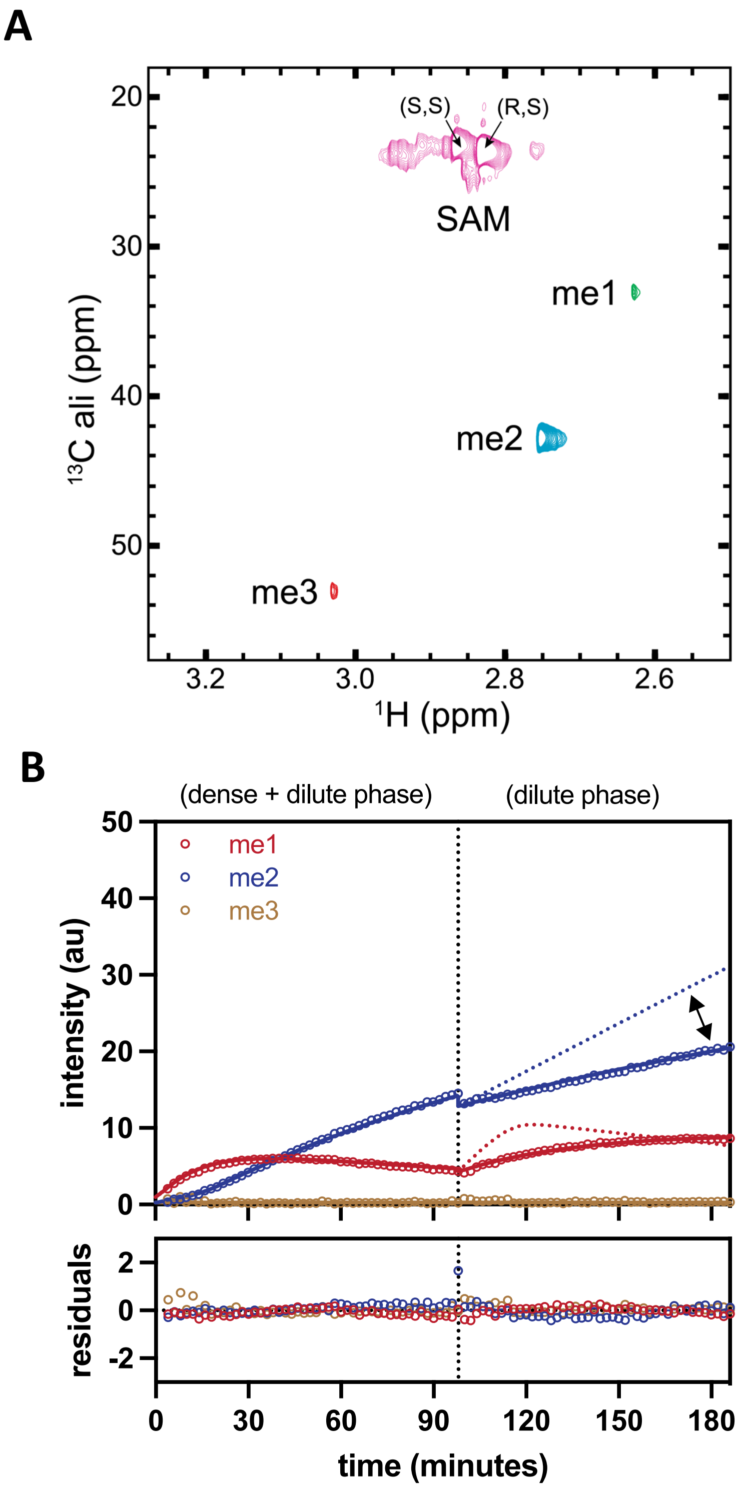

Figure S5: Methylation can be tracked in real-time with ^{13}C-SAM NMR. A. A representative spectrum from a ^{1}H, ^{13}C HSQC showing the SAM peaks (top center) and each methylation state of lysine (labeled). **B**. 200 mM NaCl reaction progress curve fit with the model in Fig. 4B.

Figure S6 – Namitz, Showalter and Cosgrove

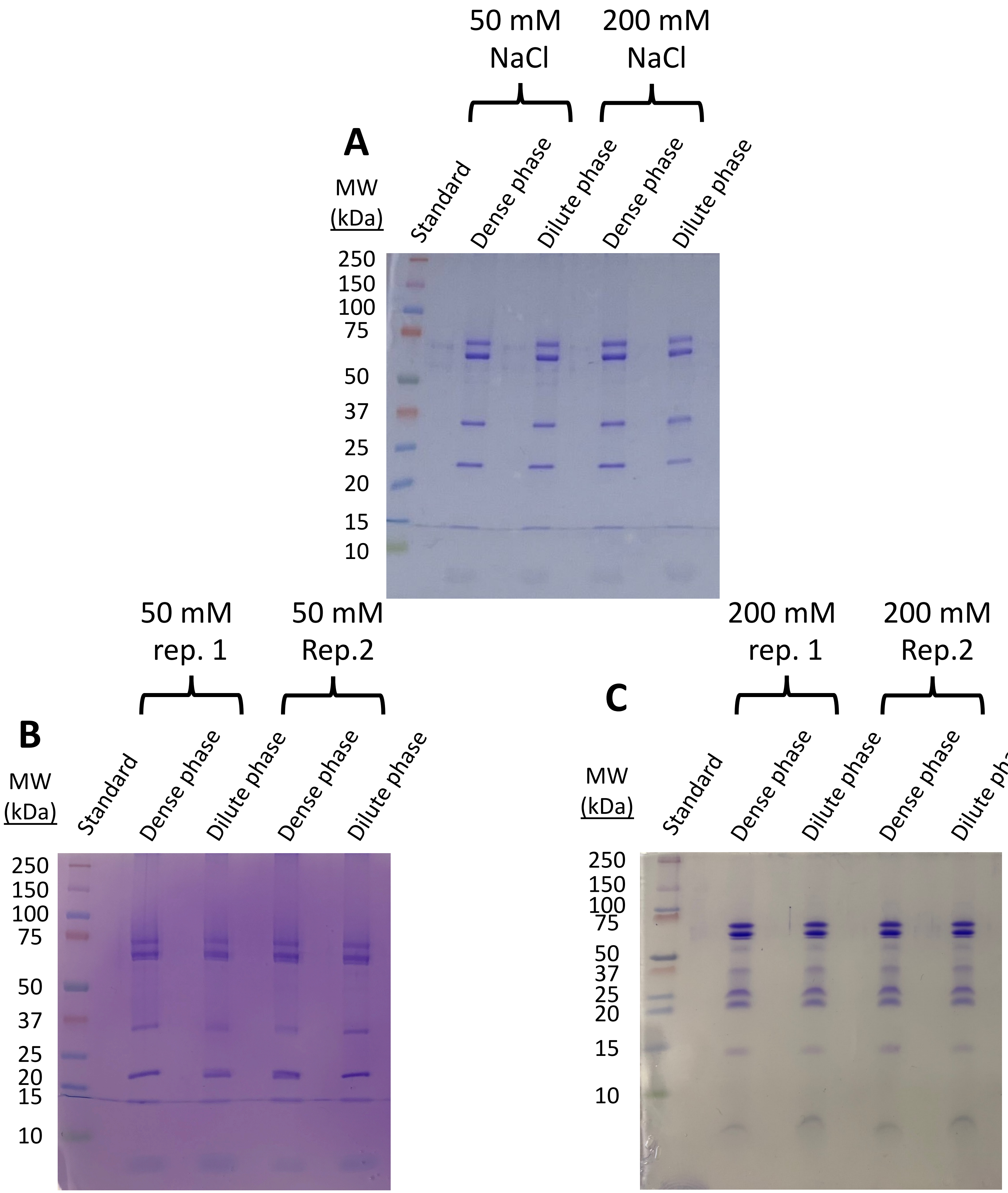

Figure S6: SDS-PAGE analysis of different phases from the MWRAD$_2$ NMR condensate activity assays. The same volume of either the dilute or dense phase of each reaction was loaded, and the intensity of the protein complex members were qualitatively compared. **A**: Gel showing 50 mM NaCl and 200 mM NaCl dense and dilute phase. **B**: Gel showing dense and dilute phases for both 50 mM NaCl replicates side-by-side. **C**: Gel showing dense and dilute phases for both 200 mM NaCl replicates side-by-side.

Figure S7 – Namitz, Showalter and Cosgrove

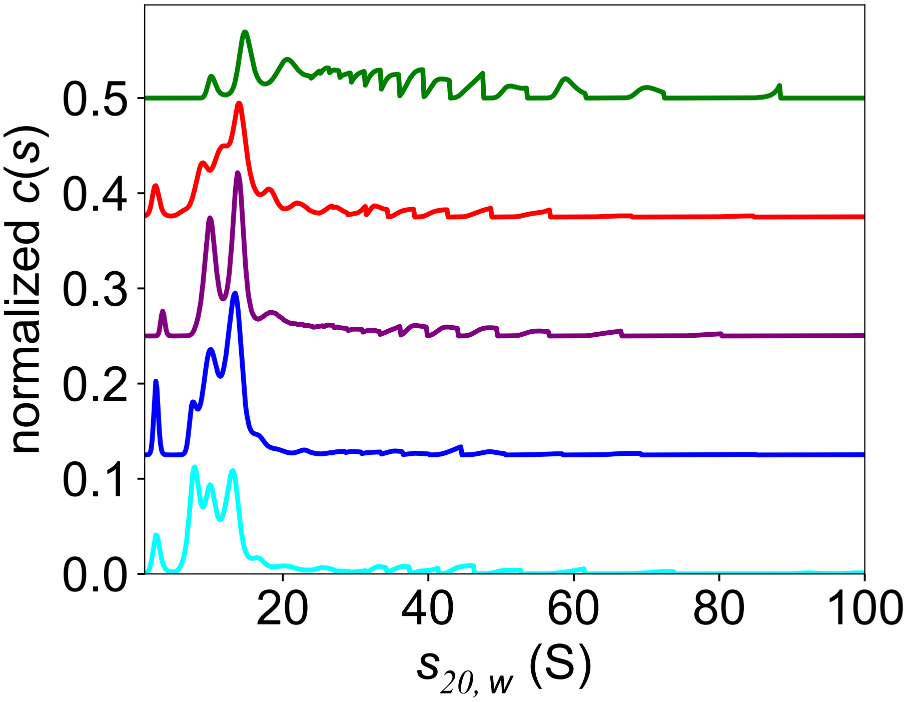

Figure S7: Concentration dependence of MLL1 core complex oligomerization at low ionic strength. MLL1 core complex was dialyzed against Tris-TCEP (with no NaCl), with three changes. SV-AUC runs were conducted with 0.25 (cyan), 0.5 (blue), 0.75 (purple), 1.0 (red) and 5.0 μM (green) $MWRAD_2$ at 25°C. c(s) plots were overlaid, and each was normalized for total integrated area.

Figure S8 – Namitz, Showalter and Cosgrove

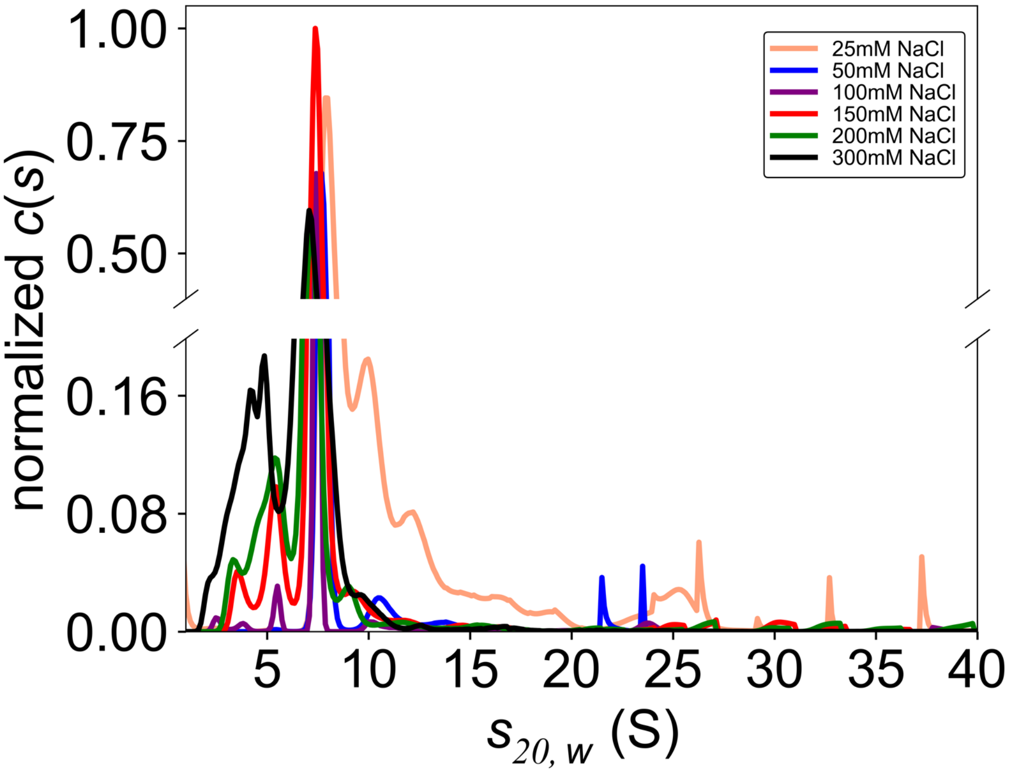

Figure S8: NaCl concentration dependence of MLL1 core complex oligomerization. SV-AUC runs were conducted with 25 (peach), 50 (blue), 100 (purple), 150 (red) 200 (green) and 300 (black) mM NaCl with 5 μM $MWRAD_2$ at 25°C. c(s) plots were overlaid, and each was normalized for total integrated area. The Y-axis was broken between 0.2 and 0.4 to aid in visualizing the higher S-value species. These data appear in separate boxes in Main-Body Figure 6A.

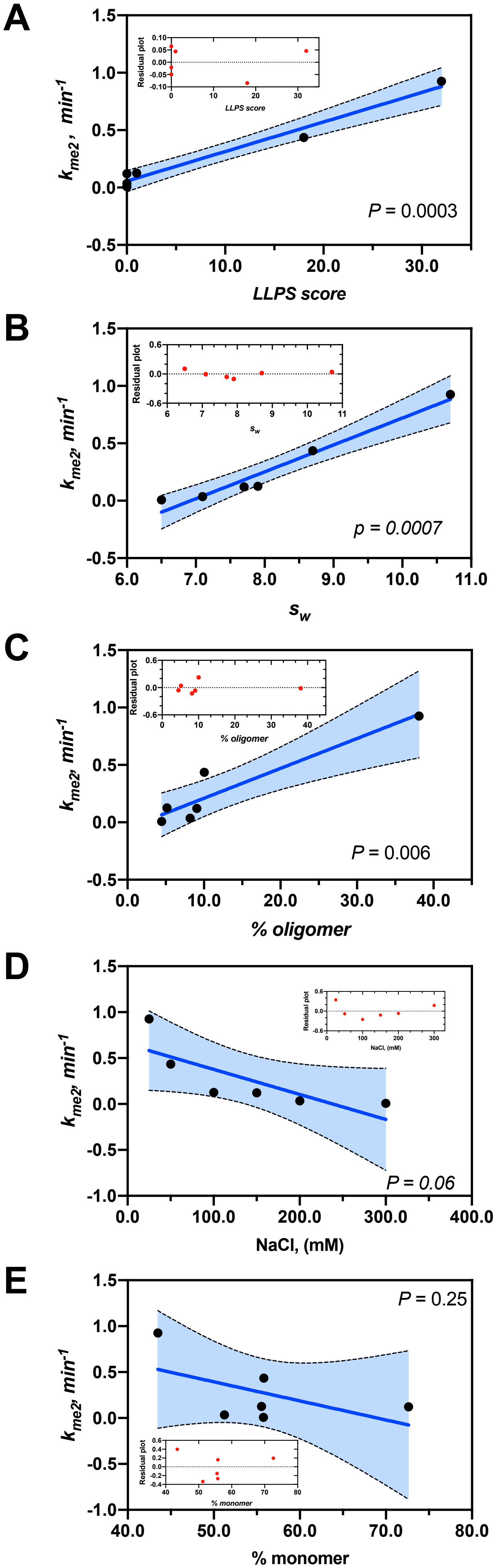

Figure S9: Increased enzymatic activity under phase separation conditions is directly related to changes in the oligomeric state of the MLL1 core complex. (A-E) Individual linear regressions of each experimental parameter on the rate of H3K4 methylation. Since the regressions were similar for each methylation event, for clarity, we display only the regressions on the rate of H3K4 dimethylation (k_{me2}, min^{-1}). For each plot, the best fit linear regression line is shown in blue with the 95% confidence interval shaded in light blue. The inset shows a plot of the fit residuals. P-values are indicated and represent the probability that the regression coefficient is different from zero by chance alone (the null hypothesis). P-values > 0.05 are considered not significant (the null hypothesis is not rejected). Linear regression of the LLPS score (A), signal weight average sedimentation coefficient (s_w) (B), % oligomer (C), ionic strength ([NaCl]) (D) and % holo-MLL1 core complex (% monomer) (E) on the rate of H3K4 dimethylation (k_{me2}) is shown. LLPS score, s_w and %oligomer show strong linear associations with the rate of H3K4 methylation with R squared values $\gtrsim$ 0.9. In contrast, Ionic strength ([NaCl]) shows a non-random distribution of residuals (R squared = 0.5-0.6) in the regression (D), indicating the absence of a direct linear relationship.

Table S1: Liquid-Liquid Phase Separation (LLPS) Prediction scores for the MLL1 core complex individual subunits, sub-complexes and $MWRAD_2$

Protein	PScore[a]	CatGRANULE[b]
MLL1	0.54	0.32
WDR5	0.11	0.44
RbBP5	0.67	0.80
Ash2L	1.80	1.07
MW	0.58	0.76
RAD_2	1.76	1.23
$MWRAD_2$	1.78	1.36

[a]: Per Vernon et al. (34), PScores >1 show possible candidates for LLPS.

[b]: Per Bolognesi et al. (35), CatGRANULE scores ~1 or larger are enriched in granule-forming (LLPS) proteins.

Table S2: Summary of the impact of phase separation on the K_m values of H3 peptide mono- and dimethylation by the MLL1 core complex.

[NaCl], mM	Phase	K_m, μM monomethyl	Fold change	Phase	K_m, μM dimethyl	Fold change
200	dilute	752.5		dilute	13.4	
	bulk	72.8	10.3	bulk	4.9	2.8
50	dilute	2285.7		dilute	163.1	
	bulk	65.0	35.1	bulk	7.5	21.9

Table S3. PCA analysis of biophysical parameters impacting the variability of H3K4 methylation activity of the MLL1 core complex. Principal Components (Eigenvectors).*

Variable	PC1	PC2	PC3	PC4
Ionic Strength	-0.406	**-0.630**	**0.514**	0.161
LLPS score	**0.488**	-0.074	0.085	**0.862**
S_w	**0.493**	0.212	0.100	-0.194
% monomer	-0.373	**0.735**	**0.518**	0.226
% oligomer	**0.464**	-0.107	**0.671**	-0.278

*Eigenvectors > 0.5 (after rounding) were considered significant and are highlighted in bold.

Principal Component	Eigenvalue	Proportion (%)	Cumulative (%)
1	3.96	79.18	79.2
2	0.68	13.58	92.8
3	0.30	5.85	98.6
4	0.07	1.39	100.0

Table S4: Liquid-Liquid Phase Separation (LLPS) Prediction scores for the SET1 H3K4 methyltransferase family

Protein	PScore[a]	CatGRANULE[b]
MLL1	4.63	1.58
MLL2	5.49	1.45
MLL3	4.64	1.37
MLL4	5.25	1.52
SETd1A	5.00	1.08
SETd1B	4.25	0.95

[a]: Per Vernon et al. (34), PScore scores ~4.0 or larger are considered strong candidates for phase separation.

[b]: Per Bolognesi et al. (35), CatGRANULE scores ~1 or larger are enriched in granule-forming (LLPS) proteins.

Table S5: Summary of the impact of ionic strength on the enzymatic, hydrodynamic and phase separation properties of the MLL1 core complex[a].

[NaCl], mM	k_{me1}, *min* $^{-1}$	k_{me2}, *min* $^{-1}$	k_{me3}, *min* $^{-1}$	s_w[b]	*LLPS score*[c]	*% monomer*[d]	*% oligomer*[e]
25	1.51	0.93	0.036	10.7	32	43.5	38.1
50	0.77	0.44	0.010	8.7	18	55.8	10.0
100	0.22	0.13	0.0007	7.9	1	55.5	5.2
150	0.22	0.12	0.0007	7.7	0	72.6	9.1
200	0.12	0.04	0.0001	7.1	0	51.2	8.2
300	0.02	0.01	0.0000	6.5	0	55.8	4.5

[a] All assays were conducted with 5 µM MLL1 core complex at 25°C in the presence of 100 µM H3 peptide and 250 µM AdoMet at pH 8.5 as described in Methods.

[b] Signal weight average s value (s_w) was obtained by integrating SV-AUC c(s) plots over the full s-value range.

[c] LLPS score = number of LLPS droplets observed in DIC microscopy images.

[d] % monomer = relative amount of absorbance signal corresponding to the holo-MLL1 core complex sedimenting between 6.8 S and 7.6 S in each c(s) plot.

[e] % oligomer is the relative amount of absorbance signal corresponding to species sedimenting between 7.6 S and 30 S in each c(s) plot.

Table S6: Sub-domain boundaries for human SET1/MLL family histone methyltransferases.[a]

(Uniprot #)	MLL1 (Q03164)	MLL2 (O14686)	MLL3 (Q8NEZ4)	MLL4 (Q9UMN6)	SETd1A (O15047)	SETd1B (Q9UPS6)
**Domain**[b]						
PHD	1431 – 1482[b]<br>1479 – 1533<br>1566 – 1627<br>1931 – 1978	170 – 218<br>226 – 276<br>273 – 323<br>1377 – 1430<br>1427 – 1477<br>1504 – 1559<br>5090 – 5137	283 – 331<br>341 – 391<br>388 – 438<br>464 – 520<br>957 – 1010<br>1007 – 1057<br>1084 – 1139<br>4460 – 4507	1201 – 1252<br>1249 – 1303<br>1335 – 1396<br>1639 – 1686		
Bromo	1703 – 1748					
AT Hooks	169 – 180<br>217 – 227<br>301 – 309		34 - 46	37 – 44<br>110 – 117<br>357 – 365		
CXXC	1147 – 1195			959 – 1006		
FYRN	2018 – 2074	5175 – 5235	4545 – 4605	1727 – 1783		
FYRC	3666 – 3747	5236 – 5321	4606 – 4691	2411 – 2492		
RRM					84 – 172	93 – 181
Win	3762 – 3767	5337 – 5342	4707 – 4712	2508 – 2513	1492 – 1497	1745 – 1750
SET	3829 – 3945	5397 – 5513	4895 – 4911	2575 – 2691	1568 – 1685	1827 – 1944
Post-SET	3953 – 3969	5521 – 5537	4895 – 4911	2699 – 2715	1691 – 1707	1950 – 1966

[a] Protein names and (Uniprot #) are listed in the topmost row.
[b]Amino acid residue range marking the beginning and end of each sub-domain, which were compiled from Uniprot.

Table S7: Sub-domain boundaries for $WRAD_2$ sub-complex members.[a]

(Uniprot #)	WDR5 (P61964)	RbBP5 (Q15291)	Ash2L (Q9UBL3-3)	DPY30 (Q9C005)
**Domain**[b]				
WD40	43 – 333	22 – 331		
Hinge		330 – 366		
PHD			23 – 56	
SPRY			266 – 489	
DD				45 – 99

[a] Protein names and (Uniprot #) are listed in the topmost row.
[b] Amino acid residue range marking the beginning and end of each sub-domain, which were compiled from Uniprot.

Supplemental Movie Legends

Movie S1: DIC microscopy movie of 5 μM $MWRAD_2$ in reaction buffer and 7% Dextran

Movie S2A and B: Macromolecular crowding induces MLL1 core complex phase separation at physiological ionic strength. DIC microscopy movies of MLL1 core complex LLPS. Macromolecular crowding with 7% dextran induces $MWRAD_2$ phase separation at low (25 mM NaCl; A) and physiological (150 mM NaCl; B) ionic strength. Arrows indicate representative fusion events indicative of LLPS.

Movie S3: Fluorescence microscopy of $M(W^*)RAD_2$ shows localization to phase condensates. Movie of $M(W^*)RAD_2$ from which Figure 9G is taken. The conditions were 5.0 μM gel filtration-purified complex (see supplementary Fig. S3) in reaction buffer with 10 μM $H3^{1\text{-}20}$ peptide, 250 μM SAM, and 150 mM NaCl.

Movie S4: Fluorescence microscopy of $MW(R^*)AD_2$ shows localization to phase condensates. Movie of $MW(R^*)AD_2$ from which Figure 9H is taken. The conditions were 5.0 μM gel filtration-purified complex (see supplementary Fig. S3) in reaction buffer with 10 μM $H3^{1\text{-}20}$ peptide, 250 μM SAM, and 150 mM NaCl.
